# How to distinguish climate sceptics, antivaxxers, and persistent sceptics: Evidence from a multi-country survey of public attitudes

**DOI:** 10.1371/journal.pone.0310325

**Published:** 2024-10-02

**Authors:** Zeynep Clulow, David Reiner

**Affiliations:** Energy Policy Research Group, Judge Business School, University of Cambridge, Cambridge, United Kingdom; Institute of Medical Biochemistry Leopoldo de Meis (IBqM) - Federal University of Rio de Janeiro (UFRJ), BRAZIL

## Abstract

Distrust in science has been linked to scepticism over vaccines and climate change. Using data from nationally representative surveys administered in eight key countries for global efforts to mitigate climate change and COVID-19 (Australia, Brazil, China, India, Japan, South Africa, the UK and US), we find that distrust in scientists was an important predictor variable for most sceptics, who were sceptical of one issue but not both, in February 2021, when most countries had experienced their first wave of the pandemic. However, the association was significantly weaker among the segment of hardcore sceptics who were both climate sceptics and antivaxxers. We demonstrate that these individuals tended to possess many of the typical sceptic characteristics such as high distrust in social institutions and rightward political orientation, which are (collectively) suggestive of an underlying sceptic mindset rather than a specific distrust of scientists. Our results suggest that different types of sceptics necessitate different strategies to dispel scepticism.

## Introduction

Distrust in science is well-known to be a key driver of climate scepticism [1–4]. People who question the scientific establishment are more likely than others to disbelieve that global warming is happening, is caused by anthropogenic activity or is having (or will have) adverse effects [5, 6]. As a result, they are less willing to adopt lifestyle changes [7] or support policies consistent with climate mitigation [5, 8–10]. Policymakers are often dissuaded from progressing climate mitigation due to the potential backlash from vested interests or sceptical segments [5, 9, 11], particularly in highly polarised contexts.

Similar concerns arise for the first novel challenge of the 2020s –the COVID-19 pandemic [12]. Compared to people who trust science, science sceptics are more likely to believe that COVID-19 poses a smaller threat [13] or originates from a different source (such as 5G technology or purposeful manufacture) [11] than that advanced by mainstream science. Attitudes towards science influence individual behaviours critical for reducing the spread of the virus such as social distancing [14, 15], compliance with self-isolation or stay-at-home mandates [16, 17] and vaccine hesitancy [18–20]. As with climate change, such sceptical attitudes could deter governments from adopting the most effective mitigative measures for combatting a pandemic if they are viewed as politically costly [11, 21–23].

Social scientists have identified several reasons why people might hold sceptical attitudes towards controversial scientific issues. The deficit model, for example, asserts that scepticism stems from a lack of accurate information [14, 24, 25]. The prevalence of misinformation about climate change and COVID-19 might be seen as explaining science scepticism on both issues [10, 13, 26–29]. However, previous research shows that information alone has limited ability to change sceptical attitudes towards climate change [30–32] and vaccines in the past, [33] suggesting it is unlikely to be the main source of scepticism [11, 19].

A better-supported explanation appears to be that scepticism towards specific domains emanates from a psychological motivation to reject science that challenges pre-existing ideological beliefs [9, 34]. For example, past studies suggest that political conservatives are more likely than liberals to be sceptics on climate change and vaccines/COVID-19 because the scientific consensus suggests remedies that are difficult to reconcile with conservative aversion to government involvement [11, 19, 35, 36].

A related thesis is that science scepticism stems from perceptions regarding the credibility of the source [4, 37–41], which is often associated with certain ideological predispositions such as conspiratorial perspectives [42], political conservatism [43] and populism [41, 44]. Believers in conspiracy theories, for example, are predisposed to distrust elites and institutions (e.g. politicians, corporations, and scientists) and, therefore, doubt their claims about scientific issues such as climate change [3, 45–47] and, more recently, the pandemic [19, 21, 22]. Similarly, populists tend to be climate sceptics because they view (climate) scientists as part of a self-serving elite that ‘betrays’ people [41]. More generally, multiple studies indicate that, across a range of ideological persuasions, distrust in scientists is strongly correlated with scepticism towards climate change [1, 41, 46, 48, 49] and COVID-19 vaccination [12, 49–51]. The association holds irrespective of access to accurate information, ideological orientation, and belief in conspiracy theories [4] and has even been found to moderate the effect of other drivers of climate beliefs such as news media [52].

There are several reasons why distrust in scientists might drive scepticism on climate change and COVID-19 vaccination. Most obviously, the complex, technical nature of both issues renders them less accessible to laypeople—trust in scientific experts becomes a cognitive shortcut for accepting mainstream understandings largely premised on scientific consensus [6, 8, 41]. Second, effective mitigation of both issues imposes significant societal costs, including radical behavioural changes such as modifying lifestyles (whether reducing carbon footprints or complying with social-distancing and lockdowns to prevent the spread of the virus), which are associated with feelings of powerlessness, creating additional impetus for questioning the scientists who inform or even champion these intrusive policy responses [11, 21, 53].

We investigate the relationship between trust in (university) scientists and sceptical attitudes towards climate change and COVID-19 vaccination among the public. Our analysis employs novel data obtained from nationally representative surveys (see Methods and Supplementary Information) administered in eight key countries for global climate change and COVID-19 mitigation (Australia, Brazil, China, India, Japan, South Africa, UK, and US). The surveys were conducted simultaneously on 22^nd^ February 2021, by which time, most countries had experienced their first national wave of the pandemic, imposed lockdowns and began rolling out mass vaccination programmes (see Sources and methods).

## Sources and methods

### Survey samples

We recruited market research company Ipsos Mori to collect data from nationally representative samples of adult populations in Australia, Brazil, China, UK, India, Japan, South Africa, and US (n = 2000 for all samples, apart from Japan where n = 2035). These countries were selected because they are responsible for important shares of (historic, present or expected future) global greenhouse gas emissions and host sizable populations which renders them critical for addressing both climate change and the pandemic. The countries also vary widely in terms of key variables in the context of this study such as trust in scientists, sceptic profiles and other potential drivers of scepticism such as poverty, natural resource abundance, political regime and other indicators of human development. This variation is important for evaluating the geographical applicability of our findings and also helps ensure that significant results reflect associations with trust in scientists rather than certain (similar) national conditions.

The surveys were conducted on the same date—22^nd^ February 2021—in all countries to minimise temporal developments relating to climate change (e.g. important climate conferences and extreme weather events) and the COVID-19 pandemic (e.g. the rollout of COVID-19 vaccination programs) that could influence attitudes towards both issues. We acknowledge that any survey can only offer a snapshot, particularly in the midst of the pandemic when different countries are at different stages of vaccine rollout. Nevertheless, we would expect that the hard core of double-sceptics would be fairly robust even if attitudes towards, say, the economy-COVID trade-off would be expected to change over time.

S3–S10 Tables in S1 File provide the demographic information for each country sample. As can be seen in these tables, our samples were balanced with respect to gender (female to male ratios were between 48 to 52%). Since respondents were recruited from people aged 18 years and above, the median age of our pooled sample was (approximately 4.6 years) older than the median age of the respective national populations. All data collection was carried out by IPSOS Mori through its own panels in compliance with the international (ESOMAR) Code of Conduct and the firm’s own Code of Conduct and Ethics, the GreenBook. As such, informed consent was obtained from each survey participant via the survey firm’s online panel methodology, which was approved by the relevant University ethics committee specified below. The study complies with all University of Cambridge and Judge Business School policies on research ethics and was approved by the Cambridge Judge Business School Departmental Ethics Review Group.

We note two important limitations of our sample. First, respondents in emerging economies were recruited from urban centres and therefore may not accurately reflect attitudes of rural populations. Second, political sensitivities prohibited us from asking Chinese respondents about political dispositions.

### Survey design

Questionnaires were translated into the native language of non-English speaking samples using professional translation services provided by Ipsos Mori and checked and, where necessary, revised by native-speaking colleagues for comprehension and translatability, particularly of technical terms. The first part of the survey asked standard demographic questions relating to the country of residence, age, educational attainment, and employment and was followed by a series of questions designed to gauge attitudes towards the environment and public health. Questions used to capture the dependent variables (scepticism towards climate change, COVID-19 vaccination and correlated attitudes towards the economy) were then presented in randomised order. Respondents rated their trust in university scientists as a subset of a longer question that captured levels of trust in several other social institutions and actors (such as corporations, national governments, television news, social media, and environmental NGOs) that were presented in random order. These questions were followed by a series of more specific questions relating to knowledge about energy and environmental issues and preferences towards a range of different climate policy options.

### Operationalisation of key variables

Respondents were asked to rate their trust in the following sources to provide accurate information on sustainable energy and environmental issues on a seven-point scale (1 = do not trust as all; 7 = trust completely or ‘don’t know’): environmental groups, oil and gas companies, Shell, Greenpeace, Television news, social media, university scientists, Greta Thunberg, and national government (excluding China). Respondents who chose the ‘don’t know’ option were treated as missing values.

We intentionally adopt strict definitions of sceptic profiles in order to focus on positions that pose the strongest opposition towards responses towards climate change and COVID-19 that are rooted in mainstream scientific understandings. Respondents were asked to rate the threat posed by climate change to their own country from the following options: a major threat, a minor threat, not a threat and don’t know. Responses were dichotomised so that people who answered that climate change did not pose a threat to their country were coded as climate sceptics (1) while those who perceived a minor or major threat were non-sceptics (0). Don’t know responses were treated as missing values.

Respondents were asked how likely they were to take a COVID-19 vaccine if offered one. Responses were recorded on a seven-point scale from 1 = already taken it/ would definitely take it to 7 = would definitely not take it. Responses were coded as a binary variable so that antivaxxers (those who answered 7) were recorded as 1 and all other responses (1–6) were coded as 0. Don’t know responses were treated as missing values. Despite our restrictive sceptic profiling, post hoc sensitivity analysis presented in the Discussion Section demonstrate that sceptic sampling was of sufficient size to conduct reliable analyses about the relationship with trust in scientists.

Full details of the data source, coding strategy and number of missing values of all variables and number of missing values included in the analyses are given in the Supplementary Information (S2 Table in S1 File and Operationalisation of Control Variables). Observations with missing values were excluded from the analysis.

## Results

### Descriptive statistics

Existing research on the relationship between trust in scientists and climate scepticism [1, 3, 54, 55] and vaccine hesitancy [1, 56] focuses mainly on polarised national contexts (notably the US and Australia) but largely omits other countries that are increasingly important for international responses to both challenges [57]. As a corrective, we used large representative national surveys (n = 2000) spanning a range of contexts with divergent levels of polarity to (separately) ask respondents about their views towards a core tenet of climate scepticism (how big a threat climate change is for their country) and antivaxxism (how likely they are to take a COVID-19 vaccine if offered one). We also asked respondents how much priority should be given to the economy vis-à-vis combatting climate change or COVID-19, which was correlated (to varying degrees across countries) with sceptical attitudes towards climate change and COVID-19 vaccination respectively. We distinguish between respondents who are sceptics on both climate change and COVID-19 vaccination (persistent or double sceptics), climate sceptics who are not antivaxxers, and antivaxxers who are not climate sceptics.

While most respondents accepted the scientific consensus on climate change and COVID-19 vaccination, many respondents exhibited at least some degree of scepticism: 35% (n = 6634) did not consider climate change a major threat to their country and 17% (n = 1747) were unlikely to take a COVID-19 vaccine if offered one. Only a small minority (1.4%, n = 203) of respondents selected the most sceptical response towards both issues, i.e., that climate change does not pose any threat to their country *and* that they would definitely not take a COVID-19 vaccine.

**Fig 1:**
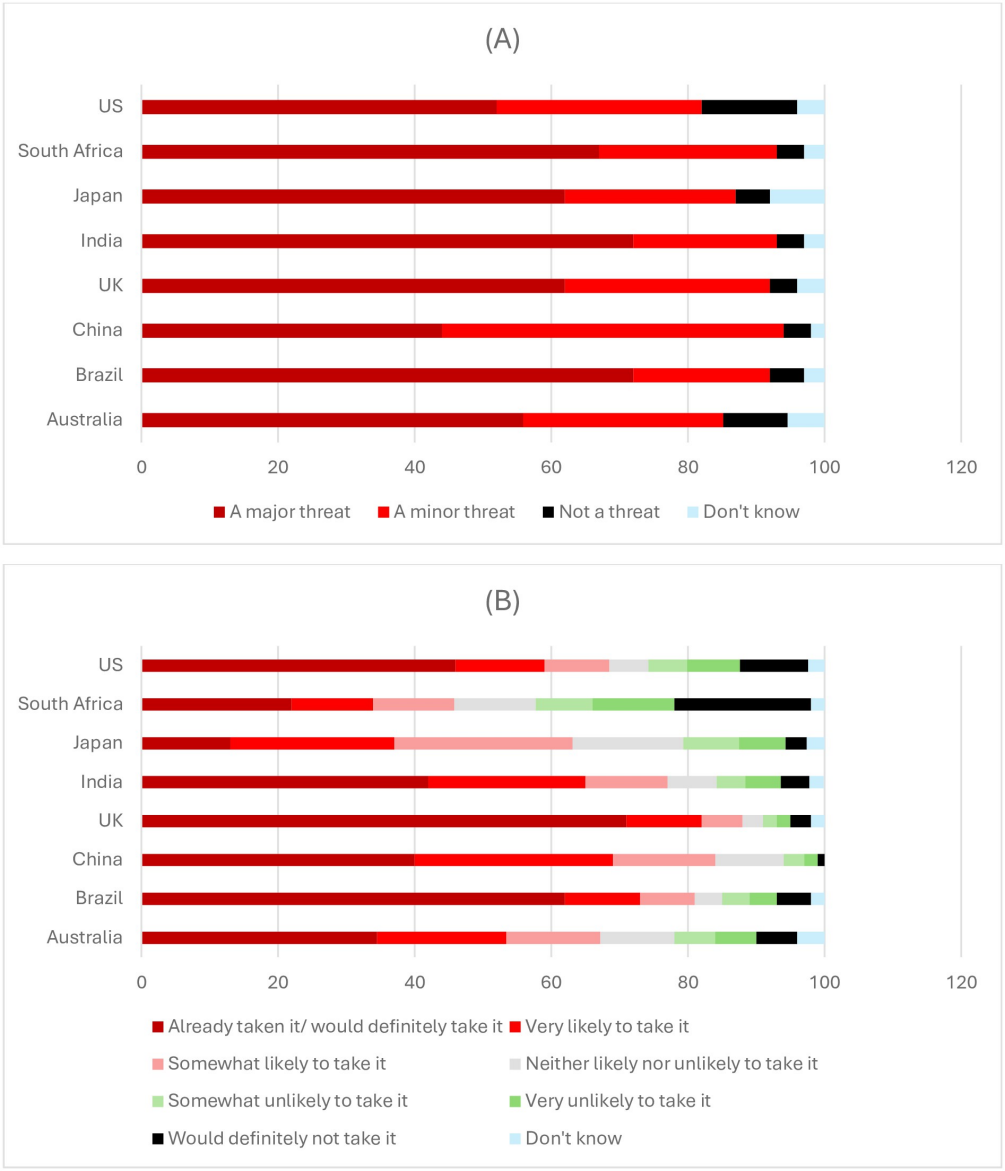
Prevalence of attitudes towards (A) climate change and (B) COVID-19 vaccination across countries. S23 Table in S1 File shows the number of respondents that selected the different Likert responses shown in Fig 1.

Less than 5% (n = 990) of respondents in 6 of the 8 countries completely dismissed the threat of climate change, but the percentage share was notably higher (P<0.01) in Australia (9%, n = 189) and the US (14%, n = 280) where public opinion on climate science is more polarised compared to other countries (Fig 1A). In 6 of the 8 countries, only a small minority (~2%, n = 1047) indicated that they would definitely not take a COVID-19 vaccine whereas fully 20% (n = 400) of South Africans and 10% (n = 200) of Americans exhibited the strongest antivaccine position (P<0.001) (Fig 1B). The number of respondents whose survey responses corresponded with the specified sceptic profiles (and equivalent economic prioritisations) are shown separately for each country in S3–S10 Tables in S1 File, and summarised in S23 Table in S1 File to aid comparison across the different country samples.

We also explored assessments of how much importance should be given to the economy over (separately) protecting the public from climate change and COVID-19. These attitudes were positively correlated with our dependent variables; pooled correlations were 0.24 for economic attitudes and climate sceptics and 0.20 for economic attitudes and antivaxxers (both P<0.001). Correlations were generally stronger in advanced economies and weaker in industrialising countries. All correlations were significant at P<0.01 or lower for all countries except for antivaxxers in China (Table 1).

**Table 1:** Pairwise correlation matrix of the dependent variables.

Sample	Climate sceptic—economy over climate change	Antivaxxer—economy over COVID-19
Pooled	0.24***	0.20***
Australia	0.37***	0.17***
Brazil	0.18***	0.23***
China	0.08***	-0.01
UK	0.34***	0.23***
India	0.07**	0.07**
Japan	0.35***	0.19***
South Africa	0.07**	0.18***
US	0.44***	0.30***

Note: Entries are Pearson’s correlation coefficients for climate sceptics and giving complete priority to the economy over combatting climate change and antivaxxers and giving complete priority to the economy over combatting COVID-19.

*** denotes p<0.001,

** p<0.01 and

* p<0.05.

### Main results

We use logistic regression to evaluate the extent to which, if at all, distrust in scientists predicts whether an individual is a climate sceptic, antivaxxer or both. Trust in scientists was positively correlated (p<0.001) with trust in national government and television news in the pooled dataset and all separate country samples except Brazil, where it was inversely correlated with trust in national government and China, where respondents were not asked about trust in government (S1 Table in S1 File). Several controls were included to isolate the association between different sceptic profiles and trust in scientists from other correlates of sceptical attitudes identified in the literature—namely: age, gender, education, prioritising environment/ health, objective climate knowledge, self-declared energy knowledge, self-declared economic hardship, responsibility attribution for resolving climate change/ COVID-19 and precautionism (a general preference for taking immediate action to prevent potentially serious societal problems versus waiting for more certain information). The results of alternative (hierarchical) regressions, which account for country-level clustering by nesting individuals within countries and include the full set of predictors (i.e. trust in other social institutions and political orientation), suggest that several of these factors are indeed important correlates of climate scepticism, antivaxxism and more generalised distrust of scientists (see Table 2): in all three domains, scepticism is inversely related with education, energy science knowledge, perceived responsibility for combating climate change and a precautionary preference for solving societal problems and, by contrast, higher among men and people who distrust television and identify with the right of the political spectrum.

**Table 2:** Associations between potential drivers of scepticism and climate scepticism, antivaxxism and distrust in scientists.

Parameter	Climate scepticism	COVID vaccine scepticism	Distrust scientists
*Fixed effects*			
Age	2.00E-3***	-0.01***	2.10E03**
Female	-0.08***	0.07*	-0.16***
Degree	-0.02T	-0.21***	-0.21***
Prioritise environment	-0.27***	-0.15***	-0.36***
Prioritise health	-0.10***	-0.29***	-0.16***
Objective knowledge	-0.03***	-0.03**	-0.08***
Self-declared energy knowledge	-3.00E-3	-0.04*	-0.08***
Perceived income insufficiency	-0.02**	-0.17***	0.03*
Climate responsibility	-0.01**	-0.04***	-0.01*
COVID-19 responsibility	-0.01*	0.01	-0.05***
Precautionism	-0.01***	-0.02**	-0.04***
Trust government	3.40E-3	-0.10***	-0.12***
Trust oil and gas comp.	0.04***	0.04***	-0.01
Trust television	-0.05***	-0.16***	-0.27***
Trust scientists	-0.08***	-0.21***	-
Left-right orientation	0.02***	0.05***	0.09***
*Random effects*			
Country variance	0.01***	0.29***	1.53***
Country-year variance	0.28***	2.94***	1.53***
R2 equivalent	0.22	0.10	0.27
LR test	323.24***	800.34***	224.75***
N	9802	9895	9992

Note: The dependent variables are continuous, climate scepticism ranges from 1 (climate change does not pose a threat to my country) to 4 (climate change poses a major threat to my country); antivaxxism from 1 (very unlikely to take a COVID vaccine if offered) to 4 (would definitely take a COVID vaccine if offered) and trust in scientists ranges from 1 (do not trust at all as a source of accurate information on sustainable energy and environmental issues) to 7 (trust completely). Models are two-level hierarchical regressions (individuals nested in countries) with random intercepts fitted using Stata’s xtmixed command. TP<0.10,

*P<0.05,

**P<0.01 and

***P<0.001.

Adj. R2 values are the square of the Pearson correlation coefficient.

S2 Table in S1 File shows the coding strategy for all variables and justification with reference to relevant literature is discussed in the SI (Operationalisation and Coding). Descriptive statistics for each country are shown in S3–S10 Tables in S1 File.

The predicted marginal effects, which denote the influence of the main predictors on the probability of holding sceptic attitudes towards climate change and COVID vaccination either separately or simultaneously, are shown in Table 3 (relative odds estimates are reported in S11, S12 Tables in S1 File). We employ separate regressions to evaluate the drivers behind three combinations of sceptical positions–(i) both climate sceptic and antivaxxer; (ii) climate sceptic but not antivaxxer; and (iii) antivaxxer but not climate sceptic—allowing us to partition our analysis to examine the role of distrust in scientists (and other factors) in predicting these different sceptical attitudes. Models 1A-3A show whether and, if so, to what extent, the explanatory variables are associated with a change in the probability that respondents are climate sceptics or antivaxxers (or both). Respondents who distrust scientists are significantly more likely to be both climate sceptics and antivaxxers compared to those who trust scientists (P<0.001). They are also significantly more likely to feel that climate change does not pose a threat to their own country without being antivaxxers (P<0.001) or feel strongly about not taking a COVID-19 vaccine without being climate sceptics (P<0.001). Similarly, models 1B-3B show that respondents who give complete precedence to the economy (whether only relative to climate or COVID-19 mitigation or both) are significantly more likely to distrust scientists (P<0.001).

**Table 3:** Effect sizes of key variables on the probability that an average individual is a: (A) climate sceptic, antivaxxer or both (N = 14956) and (B) gives complete priority to the economy over climate protection or combatting COVID-19 or both (N = 14956).

Parameter	Model A			Model B		
	1	2	3	1	2	3
	Climate sceptic and antivaxxer	Climate sceptic only	Antivaxxer only	Prioritise economy over climate and COVID	Prioritise economy over climate only	Prioritise economy over COVID-19 only
Age	9.44E-6	4.35E-4***	-1.35E-4T	6.93E-5*	4.65E-4***	1.08E-5
Female	-6.41E-4	-0.02***	0.01*	-3.07E-4	-3.20E-3	-4.12E-4
University Degree	-1.49E-3*	-2.51E-3	-0.01***	1.60E-3	-0.01**	-3.70E-3*
Prioritise health	-2.91–3***	-0.01***	-0.01**	-0.01***	-0.01**	-0.01***
Prioritise environment	-4.42***	-0.04***	-0.01	-0.01***	-0.03***	-2.49E-4
Objective knowledge	-1.19E-4	-4.58E-3***	-5.92E-4	-1.50E-3***	-0.01***	7.09E-3
Self-assessed energy knowl.	3.99-ET	3.21E-3**	3.43E-4	0.01***	0.01***	2.02E-3*
Trust in scientists	-1.51E-3***	-0.01***	-0.01***	-2.14E-3***	-3.87E-3***	-2.32E-3***
Perceived income sufficiency	1.16E-4	-9.44E-4	0.01***	3.00E-4	4.00E-3**	1.70E-3*
Climate responsibility	-7.32E-6	-2.94E-3***	1.85E-3*	-1.21E-3***	-3.50E-4	-2.53E-4
COVID-19 responsibility	-4.37E-4***	9.33E-4*	-2.72E-4	-1.81E-3***	1.29E-3T	-2.01E-3***
Precautionism	1.27E-4	-1.46E-3***	1.46E-3**	0.01***	4.23E-3***	1.28E-3***
N	14956	14956	14956	14956	14956	14956
No. of positive outcomes	203	796	816	469	867	388
R2	0.27	0.16	0.14	0.23	0.08	0.10

Note: In Models 1A-3A, the dependent variable is binary, taking the value of 1 if an individual response is categorised as the defined sceptic attitude towards climate change and COVID-19 and 0 otherwise. Model 1A estimates the probability of an individual being both climate sceptic and antivaxxer, model 2A climate sceptic but not antivaxxer and model 3A antivaxxer but not climate sceptic. In Models 1B-3B, the dependent variable is binary, taking the value of 1 if an individual response is categorised as the defined sceptic attitude towards climate change and COVID-19 and 0 otherwise. Model 1B estimates the probability of an individual giving complete priority to the economy over combatting climate change and the pandemic, model 2B the probability of giving complete priority to the economy over climate protection but not combatting COVID-19 and model 3B the probability of giving complete priority to the economy over combatting COVID-19 but not climate protection. Coefficients are marginal effect sizes that describe the probability that an average individual holds a specified sceptic (or associated economy-prioritising) profile as a result of a change in the independent variable. Marginal effect sizes are based on conversations of the effects of the independent variables on the relative log odds of the sceptic profiles (reported in S11, S12 Tables in S1 File). Country controls are included but not reported. Individual country regressions are reported in S18, S19 Tables in S1 File. ‘No. of positive outcomes’ denotes number of respondents who possess the sceptic attitude (or corresponding prioritisation of the economy) captured in the dependent variable.

*P<0.05,

**P<0.01 and

***P<0.001.

In this study, all reported R2 values are McFadden Pseudo R2s unless stated otherwise.

The differences between the effect sizes of the trust estimates across the models suggest that the association with trust in scientists varies widely between different sceptic profiles. The trust estimates are identical (0.01) in the climate sceptic (2A) and antivaxxer (3A) models; strikingly, around ten-times the size of the trust estimate in the double sceptic (1A) model—a strong indication that trust in scientists is more closely correlated with single-issue sceptics and, therefore, a better predictor of single-issue sceptic profiles, compared to double-issue sceptics. The relatively large (absolute) effect size of trust in the single-issue sceptic models (second only to environment prioritisation and gender in Model 2A and one of the largest coefficients in Model 3A), suggests that distrust in scientists is a central predictor of single-issue sceptics (estimates relating to other independent variables are discussed in the SI). Though the divergence between trust estimates across the economy prioritiser models (1B-3B) is not as wide, consistent with the sceptic profile models, the double economy prioritiser model (B1) exhibits the weakest trust coefficient of the three models, further suggesting that distrust in scientists is a better predictor of sceptical attitudes towards climate change and COVID-19 separately than both issues simultaneously.

**Fig 2:**
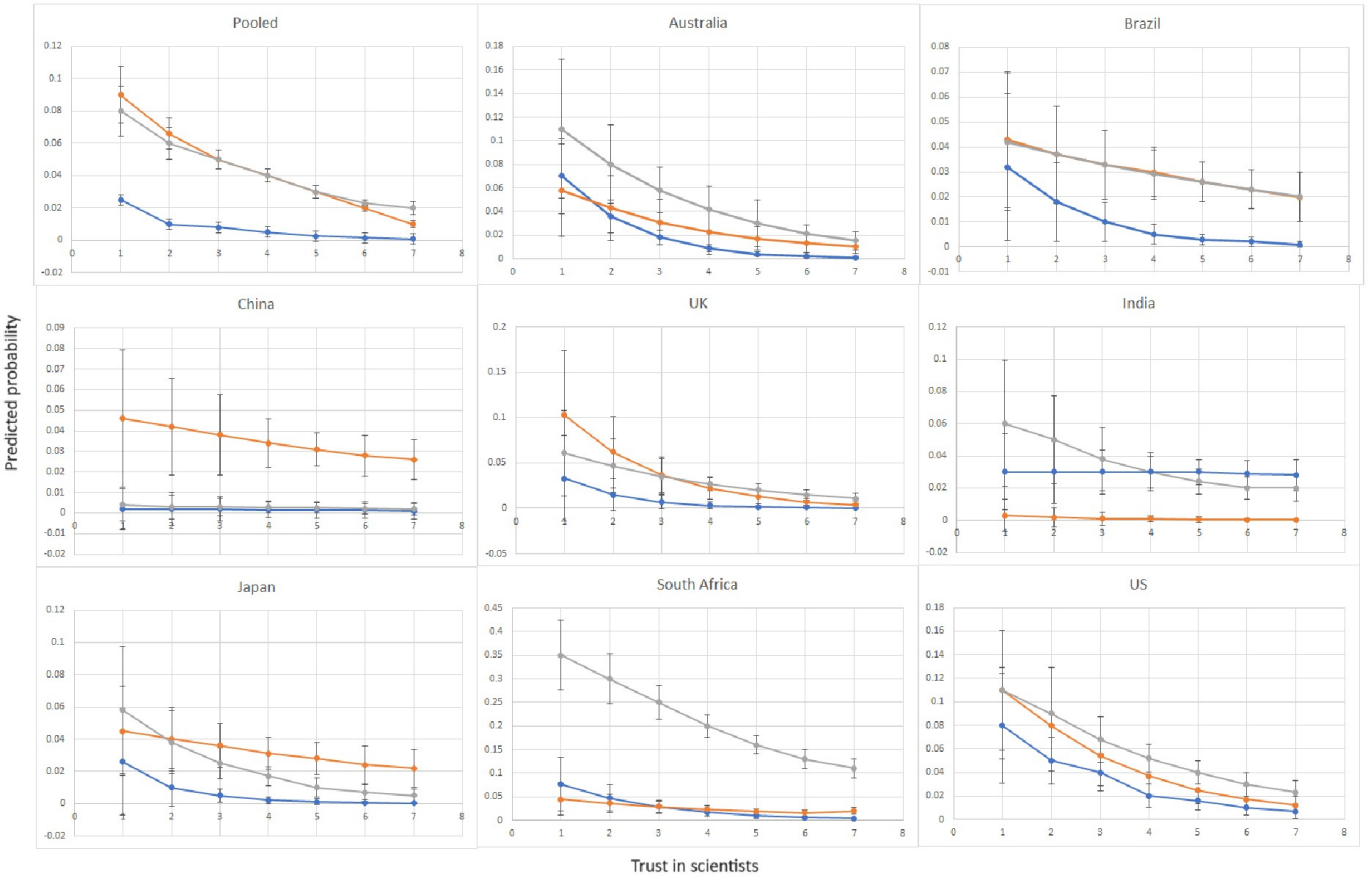
Predicted probability of being a double sceptic, climate sceptic or antivaxxer depending on trust in university scientists in the pooled and separate country models. Note: The probabilities of holding a sceptical attitude are predicted by the logistic regression models in Table 2 models 1A-3A (pooled sample) and S16 Table in S1 File (separate country samples). Blue lines show the difference in the predicted probability of being a double sceptic, orange lines climate sceptic and grey lines antivaxxer for respondents who have different levels of trust in university scientists. Vertical lines show the 95% confidence intervals.

Yet, as the estimates measure the mean association between trust and sceptical attitudes, they conceal important nuances in the association between trust and sceptic profiles across different levels of trust. Fig 2 addresses this limitation by showing how the probability of holding different combinations of sceptic attitudes varies depending on the level of trust in university scientists (as reflected by responses along a 7-point Likert scale) in the pooled and separate country samples. In the pooled sample, across all trust levels, distrust in scientists is consistently a stronger predictor of being a single-issue rather than a double sceptic, suggesting that it is a much weaker correlate of sceptical attitudes among double sceptics compared to those who are only sceptics towards either climate change or COVID-19 vaccination: Strikingly, people who completely distrust scientists are approximately four times more likely to be antivaxxers and five times more likely to be climate sceptics than double-sceptics. Except for India and China, where there were important limitations to our research methodology (the exclusion of rural respondents in India and sensitive political environment in China), we observe similar patterns in the equivalent separate country probabilities.

**Fig 3:**
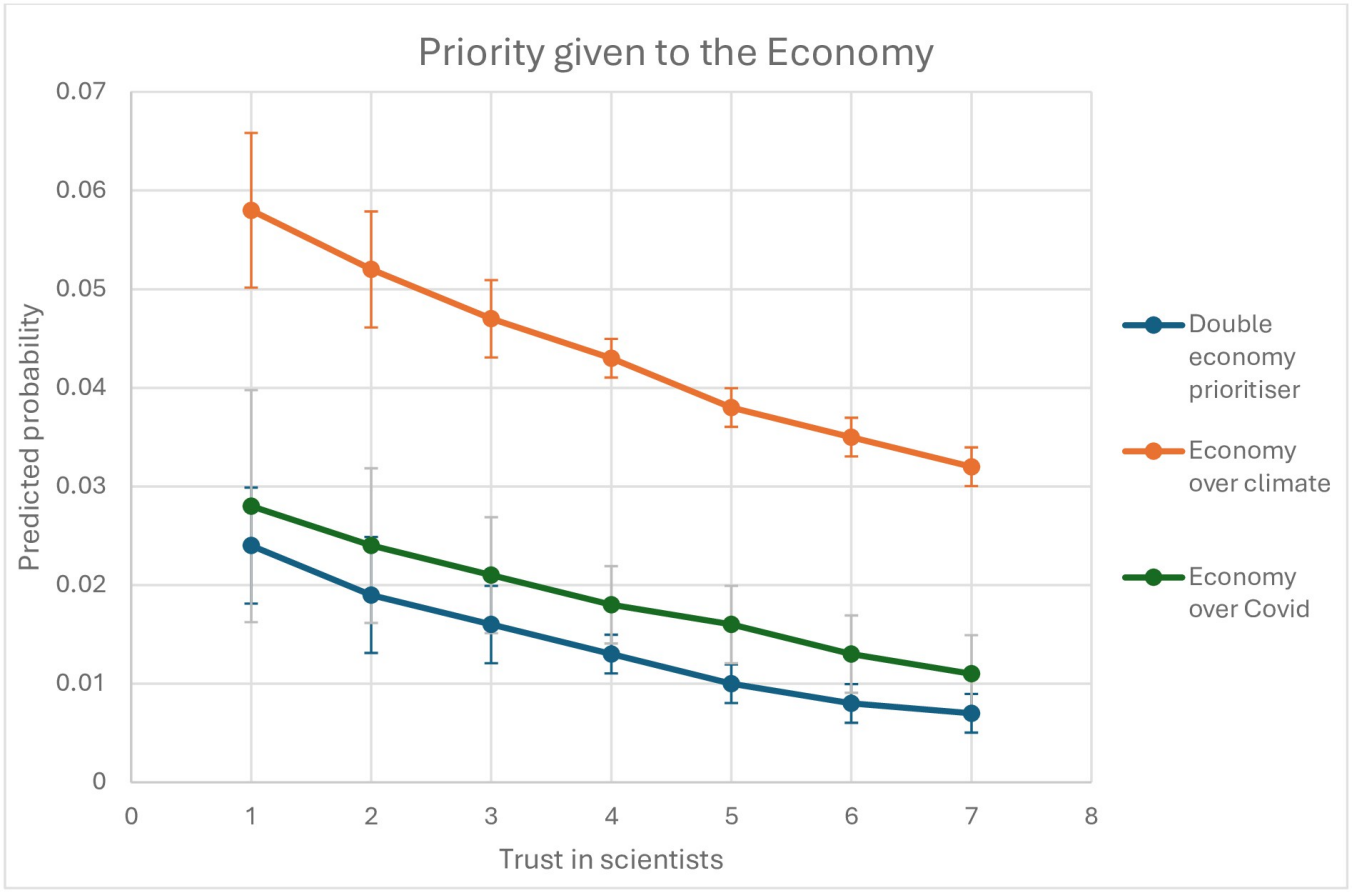
Predicted probability of giving complete precedence to the economy over climate change and COVID-19, climate change or COVID-19 depending on trust in university scientists.

Fig 3 shows that the association between trust in scientists and the probability of giving complete precedence to the economy over climate change and COVID-19 is consistently weaker than the association with people who give complete priority to the economy over the climate (but not COVID-19), providing further evidence that distrust in scientists is a good predictor of people who hold prioritisations of the economy that are compatible with climate sceptics, in contrast to respondents whose economic prioritisations align with double sceptics. However, in contrast to Fig 2, the overlapping confidence intervals of the economy-over-COVID (grey) and double economy (blue) prioritiser lines do not provide sufficient evidence to conclude that distrust in scientists strongly correlated with people who prioritise the economy-over-COVID only compared to people who prioritise the economy over both climate change and COVID-19. This could point to a limitation in our dependent variable rather than the relative weak association with scientific trust for economy-over-COVID prioritisers as preventative measures against the pandemic have resulted in more immediate economic effects compared to climate mitigation, which may have affected the priority that people give to economy relative to COVID-19.

The probability of completely prioritising the economy predicted by the logistic regression models shown in Table 3 (Models 1B-3B). The vertical lines show the 95% confidence intervals.

Our main results also hold when we replace the dependent variable with ‘making lifestyle changes to address climate change’ (S13 Table in S1 File), suggesting distrust in scientists might inhibit behavioural change compatible with climate mitigation amongst single-sceptics (model 2), while having significantly (P<0.001) weaker effects on the small group of double-sceptics (model 1). However, the relationship between lifestyle changes due to COVID-19 and trust in scientists (model 3) is notably weaker than the estimates in models 1–2 (though still significant at P<0.001)–a likely reflection of the unprecedented nature of COVID-19-related lifestyle changes imposed over 2020–21 rather than the diminished role of trust in scientists.

### The sceptic mindset is central for hardcore sceptics

Previous research suggests that people who are both climate sceptics and antivaxxers tend to hold a more intense range of attitudes such as, for example, (far-right) conservative ideological orientation [11, 12, 21, 35, 58] and general (strong) distrust of elite institutions, which are typically associated with people who are sceptics towards a broader range of issues [35, 39, 45, 46]. Therefore, a possible reason for the relatively stronger predictive power of distrust in scientists over single versus double sceptics is that the latter are motivated by a psychological need to reject scientific consensus as a way of reinforcing the underlying worldviews, ideologies and fears that comprise the sceptic mindset [12, 52, 58]. By contrast, single-issue sceptics, who do not possess such psychological bias against elite institutions, are affected by a more isolated distrust in scientific consensus on climate change or COVID-19 vaccination. While we cannot conclusively assess whether trust in scientists does indeed have the asserted causal influence over the different sceptic profiles, the strength and direction of the estimated marginal effects analysed above are in accordance with this thesis.

**Fig 4:**
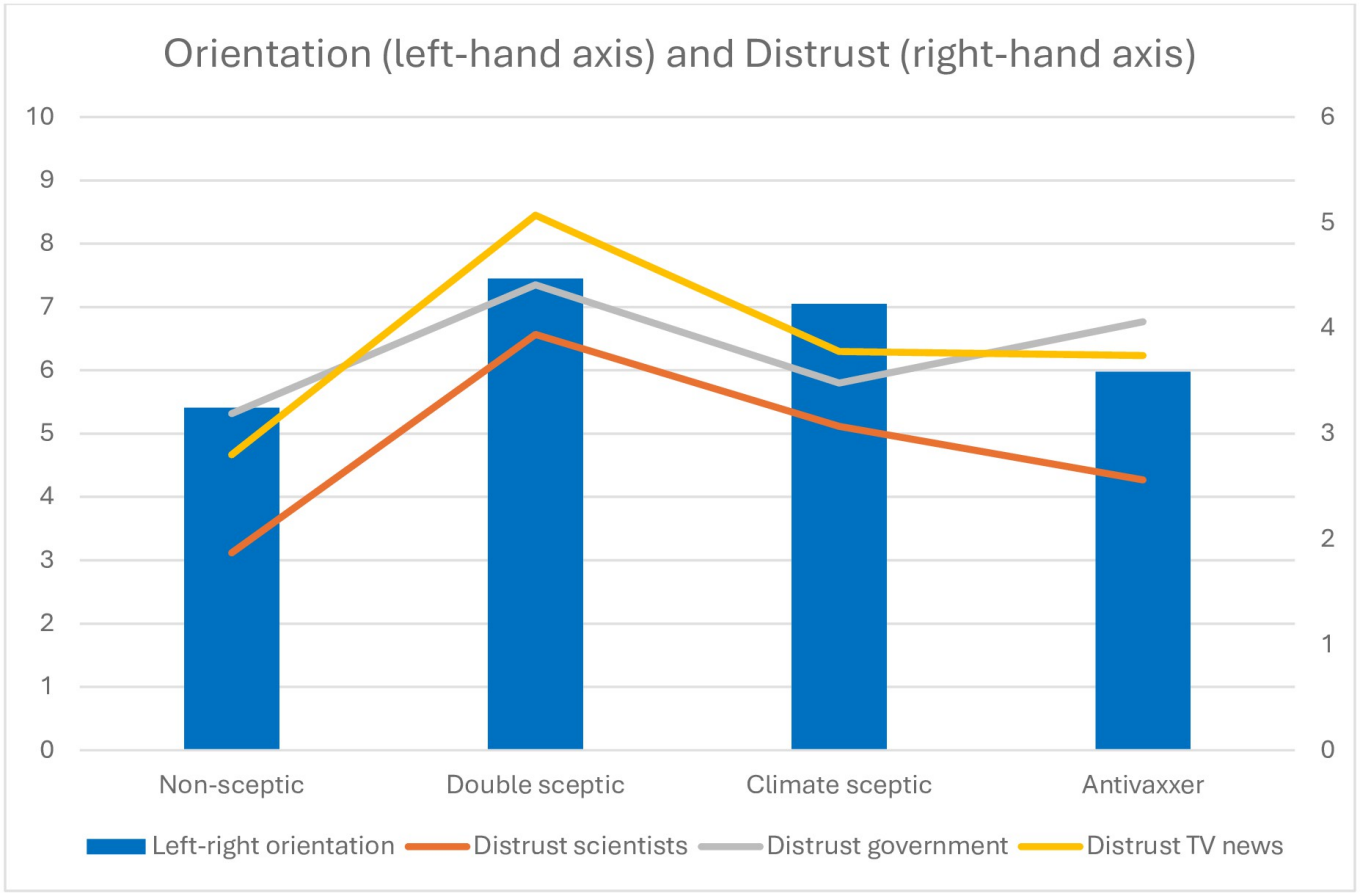
Pooled mean values of four key variables across non-sceptic, double sceptic, and single sceptic segments. Note: The blue bars show the mean score respondents assigned when asked to locate themselves on a left-right political scale from 0 (left) to 10 (right). Orange lines show mean levels of respondents’ distrust in university scientists, grey lines their national government and yellow lines television news from 0 (completely trust) to 6 (do not trust at all). Chinese respondents were not asked questions on left-right orientation and distrust in national government.

Furthermore, differences in core beliefs and attitudes that are typically associated with a sceptic worldview across the different sceptic profiles in our sample are also consistent with this claim. Fig 4 shows the pooled differences between some of the core sceptical attitudes among double-sceptics, single-sceptics (sceptics on either climate change or COVID-19 vaccination) and non-sceptics. Except for a (6%) minority of antivaxxers on the far-left (corresponding to 0 on our 0–10 Likert scale) of the political spectrum, respondents who fall under any of the three sceptic combinations are generally more right-leaning and distrusting of social institutions compared to non-sceptic respondents (S14, S15 Tables in S1 File). Yet, apart from the far-left antivaxxer minority, people who are both climate sceptics and antivaxxers tend to situate themselves further right-of-centre and exhibit higher levels of distrust towards scientists, their national government and television news compared to single-sceptics. Differences in political orientation and distrust in social institutions are consistently significant (P<0.001) between double and single sceptics, and between non-sceptics and (double and single) sceptics (S14, S15 Tables in S1 File). These patterns hold true for most of the separate country samples (S1 Fig in S1 File): In Australia, the UK, US and South Africa, double sceptics tend to be further right of centre and express higher distrust in national government and television news compared to single sceptics. In India and Japan, distrust in government and television news (but not rightward orientation) is higher among double sceptics than single sceptics and, in Brazil, distrust in television news (but not national government or political orientation) is higher among double sceptics. In China, where we did not ask about trust in government or political orientation, double sceptics exhibit higher distrust in television news compared to single sceptics.

More sophisticated pooled regressions that account for country-clustering with a hierarchical design indicate that the (absolute) size of the association between double sceptics and rightward political orientation and distrust in television—potential correlates of sceptical worldviews—is significantly stronger (two- to three-fold) than for single-issue sceptics, whereas certain demographic variables (namely: age, gender, climate responsibility and precautionary preference) are predictors of single, but not, double-issue sceptics (Table 4). Yet, contrary to the single-level model results, the marginal effects estimated by the hierarchical regressions suggest that the correlation with distrust in scientists is stronger for double sceptics than single-issue sceptics (around 30% more than climate sceptics and approximately double that of antivaxxers). Collectively, the relatively stronger correlation between double sceptics (vs. single-issue sceptics), rightward political orientation and distrust of elite social institutions lend credit to the thesis that hardcore sceptics are motivated by a generalised sceptical worldview rather than a more isolated distrust of scientists.

**Table 4:** Effects of key variables on the probability that an individual is a climate sceptic, antivaxxer or both in hierarchical configurations.

Parameter	Climate sceptic	Antivaxxer	Double sceptic
*Fixed effects*			
Age	0.01***	-0.01*	1.60E-4
Female	-0.76***	0.15	-0.15
Degree	0.02	-0.28*	-0.36T
Prioritise environment	-1.89***	-0.14	-1.66**
Prioritise health	-0.40***	-0.20*	-0.84***
Objective knowledge	-0.16***	-0.04	-0.02
Self-declared energy knowledge	0.06	0.02	0.16T
Perceived income insufficiency	-0.02	0.24***	0.21*
Climate responsibility	-0.10***	0.06**	0.01
COVID-19 responsibility	0.04T	-0.01	-0.17***
Precautionism	-0.05**	0.04*	0.01
Trust government	-0.03	-0.14***	-0.10
Trust oil and gas comp.	0.25***	8.90E-4	0.05
Trust television	-0.19***	-0.21***	-0.62***
Trust scientists	-0.27***	-0.17***	-0.35***
Left-right orientation	0.08***	0.05**	0.11**
*Random effects*			
Country variance	0.22***	0.39***	0.34***
R2 equivalent	0.01	0.56	0.70
LR test	54.91***	188.89***	11.54***
N	9992	9992	9992

Note: The dependent variable is binary, taking the value of 1 if an individual response is categorised as fitting the specified sceptic profile and 0 otherwise. Models are two-level hierarchical logistic regressions (individuals nested in countries) with random intercepts fitted using Stata’s xtmelogit command. In accordance with established methods for evaluating the goodness of fit of multilevel models [59], (country level) null variance components were used to calculate the percentage of explained variance at the country level and reported as the R2 equivalent. Marginal effect sizes are based on conversations of the effects of the independent variables on the relative log odds of the sceptic profiles shown in S20 Table in S1 File. T denotes P<0.10,

*P<0.05,

**P<0.01 and

***P<0.001.

When the far-left and far-right (respectively corresponding to 0 and 10 on our Likert scale) are coded as separate (binary) variables rather than on an interval scale, the correlation between far-right orientation and double sceptics (significant at P<0.001) is stronger than the correlation with people who are either climate sceptics (but not antivaxxers) or antivaxxers (but not climate sceptics) in the pooled and separate Brazil and UK samples (Table 5). In Australia and the US, where climate change is more polarised, the correlation between double sceptics and the far-right is stronger than the correlation with antivaxxers, but not climate sceptics. In Japan and the US, we also find significant (P<0.05 and <0.001 respectively) inverse correlations between the far left and double (but not single) sceptics, providing further tentative evidence that, at least at the extremes, political orientation is associated with double, but not, single sceptics. While the correlation coefficients are relatively small in magnitude, Pearson’s parametric correlation tests show that most (all except double sceptics in China and Japan and antivaxxers in China) of the correlations between trust in scientists and sceptic profiles attain or approach statistical significance. However, comparisons of the correlation coefficients across double and single-issue sceptic profiles find little evidence of significant differences within the separate country samples (S16 Table in S1 File). Yet the lack of significant differences could reflect the relatively small number of respondents that fit the sceptic profiles in the separate country samples rather than an indication of uniform correlation (see S17 Table in S1 File for the numbers of sceptic respondents in the pooled and separate country samples). Indeed, the results of the pooled comparisons indicate that the correlation with trust in scientists is significantly (at P<0.05) weaker for double sceptics than climate sceptics (S16 Table in S1 File), providing support for the latter perspective.

**Table 5:** Pairwise correlation matrix of sceptic attitudes and key sceptic attributes.

Sample	Model A—Double sceptic					Model B—Climate sceptic					Model C—Antivaxxer				
	(1) Scientists	(2) TV news	(3) Govt	(4) Far left	(5) Far right	(1) Scientists	(2) TV news	(3) Govt	(4) Far left	(5) Far right	(1) Scientists	(2) TV news	(3) Govt	(4) Far left	(5) Far right
Pooled	-0.02***	-0.14***	-0.08***	-0.01	0.09***	-0.16***	-0.11***	-0.03**	-0.02**	0.07***	-0.09***	-0.11***	-0.11***	0.03***	0.05***
Australia	-0.21***	-0.15***	-0.07*	-0.02	0.14***	-0.23***	-0.10***	0.04T	-0.03	0.15***	-0.12***	-0.12***	-0.12***	0.04T	0.04
Brazil	-0.14***	-0.13***	0.11***	0.01	0.15***	-0.08**	-0.03	0.09***	-0.03	0.08**	-0.11***	-0.09***	0.10***	-0.03	0.12***
China	-3.40E-3	-0.07**	-	-	-	-0.04*	-0.05*	-	-	-	-2.00E-3	-0.04***	-	-	-
UK	-0.09***	-0.09***	-0.07	0.02	0.18**	-0.22***	-0.12***	-0.02	-0.01	0.06**	-0.08***	-0.14***	-0.13***	0.02	-0.01
India	0.07**	-0.06*	-0.09***	-0.01	4.60E-3	-0.12***	-0.01	-0.04T	-0.03	-1.40E-3	-0.04T	-0.11***	-0.08***	0.03	0.03
Japan	-0.11***	-0.09**	-0.08***	-0.01***	-0.01	-0.04T	-0.12***	0.01	0.01	0.10***	-0.11***	-0.14***	-0.10***	0.02	0.02
South Africa	-0.13***	-0.11***	-0.09***	-3.70E-3	0.01	-0.07**	-0.04T	-0.02	-0.01	-0.02	-0.16***	-0.15***	-0.18***	0.06*	0.02
US	-0.25***	-0.22***	-0.17***	-0.05*	0.20***	-0.34***	-0.22***	-0.17***	-0.05*	0.22***	-0.11***	-0.11***	-0.15***	-0.03	0.03T

Note: Entries are Pearson’s correlation coefficients between double sceptics, climate sceptics and antivaxxers and key sceptic attributes. Chinese respondents were not asked to rate trust in government or political orientation.

*** denotes p<0.001,

** p<0.01,

* p<0.05 and T p<0.10.

We observe stronger correlations between double sceptics and their trust (or rather distrust) in societal institutions, which coheres with our proposition that a minority of hardcore sceptics who reject the scientific consensus on both climate change and COVID-19 vaccination are motivated by an underlying sceptic mindset, in contrast to single-issue sceptics, whose scepticism appears to be relatively autonomous of most key sceptic attitudes apart from trust in scientists. For example, double sceptics are less trusting of television news than climate sceptics and antivaxxers in the pooled dataset and separate Australia, Brazil, and China samples, (column 2, model A vs. models B&C) and climate sceptics (but not antivaxxers) in India and South Africa and antivaxxers (but not climate sceptics) in the US. Similarly, in Brazil and India, the correlation between trust in government and double sceptics is stronger compared to the equivalent correlations with climate sceptics (column 3, model A vs. model B) and, in the pooled sample and Australia, Japan and South Africa and the US, trust in government is more strongly correlated with double sceptics than antivaxxers (column 3, model A vs. model C).

When we include trust in television news, national government, and political orientation as predictors in our core regression, the association between double sceptics and trust in scientists continues to be significantly weaker (P<0.001) compared to associations with most other key sceptic attitudes (column 1 vs. columns 2–6, model A) in most subsets of our dataset (Table 6). Compared to the climate sceptic (Model B, column 1) and antivaxxer (column 1, model C,) models, trust in scientists is associated with a smaller (absolute) coefficient in the double sceptic model (column 1, model A) in the pooled sample and most countries, providing further indication that trust in scientists has a weaker relationship with double sceptics compared to both types of single-issue sceptic (as is the case for the pooled model) or climate sceptics (as in the UK, India and the US) or antivaxxers (as in Australia, Brazil and South Africa) separately.

**Table 6:** Probability of being different sceptic attitude combinations depending on key sceptic attributes in the pooled and separate country samples.

Country	(1) Trust in scientists	(2) Trust in TV news	(3) Trust in govt.	(4) Left-right	(5) Far-left	(6) Far-right
*Model A—Double sceptic*						
Pooled	4.20E-3***	-0.01***	7.75E-4	1.44E-3***	-2.56E-3	0.01***
Australia	-0.01**	-0.02**	1.42E-3	3.78E-3	-0.01	0.02*
Brazil	-6.02E-3**	-0.01**	4.87E-3	1.55E-3	0.02	0.01
China	0.01	-0.01	-	-	-	-
UK	-1.56E-3	3.39E-3	-3.55E-3*	3.10E-3T	X	4.34
India	-9.15E-4	X	-1.70e-3	2.98E-3	X	-3.03E-3
Japan	1.56E-3	-0.01*	-0.06T	-1.66E-3	X	X
South Africa	-1.03E-3**	-3.58E-3	-0.01*	1.03E-3	-4.02E-3	0.01
US	-0.01T	-0.02**	-0.01T	0.01T	X	0.03**
*Model B—Climate sceptic*						
Pooled	-0.01***	-3.87E-3**	-2.65E-3*	4.95E-3***	0.01	0.02***
Australia	-0.02*	-0.03***	0.02*	0.02**	0.03	0.04*
Brazil	-3.80E-3	1.01E-3	4.50E-3	2.88e-3	4.12E-3	0.01
China	-1.34E-3	-0.01*	-	-	-	-
UK	-0.02***	-4.00e-3	2.80E-3	1.23e-3	0.03	0.01
India	-0.01**	X	3.10e-3	8.78E-3	X	3.85E-3
Japan	1.48E-4	-0.02***	0.01	4.89E-3	0.03	0.05*
South Africa	-2.58E-3	-3.91E-4	-1.80E-3	4.55E-4	-3.99E-4	-0.01
US	-0.02**	-0.01	-0.01	0.01**	0.06	0.04**
*Model C—Antivaxxer*						
Pooled	-0.01***	-0.01***	0.01***	2.43E-3**	0.02*	0.03***
Australia	-0.01**	-4.61E-3	-0.01*	-4.00E-4	0.03	0.02
Brazil	-8.89E-3**	-4.00e-3	0.01**	0.01**	-0.01	0.03**
China	4.04E-3	-2.11e-3T	-	-	-	-
UK	-2.80E-3	-0.01*	-4.80E-3	1.23E-3	-4.40E-4	-0.01
India	-1.60E-3	X	-0.01**	2.00E-3	0.01	0.03***
Japan	-4.04E-3	-0.01**	-1.86E-3	7.09E-5	X	0.01
South Africa	-0.02***	-0.01	-0.03***	1.70e-3	0.08**	0.05T
US	-0.01	-0.01T	-0.02**	2.90E-3	-0.02	0.01T

Note: The dependent variable is binary, taking the value of 1 if an individual response is categorised as the defined sceptic attitude towards climate change and COVID-19 and 0 otherwise. Model (A) estimates the probability of an individual being both climate sceptic and antivaxxer, model (B) climate sceptic but not antivaxxer and model (C) antivaxxer but not climate sceptic. Alongside the full set of control variables from Table 1, Models 1–6 include trust in scientists, TV news and national government and political orientation (coded as an interval variable from 0 to 10 in model 4 and binary far-left and far-right orientation in models 1–3 & 5–6. T denotes P<0.10,

* P<0.05,

** P<0.01 and

*** P<0.001.

X indicates missing due to collinearity.

When considered alongside our previous results in Table 5, which showed that a broader range of factors such as (self-declared) energy knowledge, perceived economic hardship and precautionism are separately associated with climate sceptics and antivaxxers but not double-sceptics, these findings suggest that single-issue sceptics are correlated with and potentially motivated by different factors than double sceptics.

## Discussion

Our analyses make important contributions to existing understandings about the relationship between trust in scientists and scepticism regarding climate change and COVID-19 vaccination. We find strong evidence that most climate sceptics and most antivaxxers, who are sceptics on one issue but not both, are related to a localised distrust in scientists, whereas, for double sceptics, scepticism appears to be more autonomous of trust in scientists and deeply rooted in underlying sceptical worldviews [12, 20, 21, 35]. This latter proposition could be tested further by exploring whether our ‘double’ sceptics also hold sceptical attitudes towards a wider range of potentially controversial scientific practices such as genetically-modified crops, nuclear energy, fracking and wind turbines and broader anti-vax sentiments or scepticism towards conventional medicine. Given that antivax attitudes have immediate personal repercussions one might also try to separate out scepticism regarding policy interventions from scepticism that impacts directly on one’s person and one might expect whether there are groupings of issues associated with what we call here single sceptics.

Our data also suggest that for the vast majority of climate sceptics and antivaxxers, i.e., those who are not sceptics on both issues, sceptical attitudes are also related to issues such as energy knowledge, perceived economic hardship and precautionism as well as distrust of scientists. The distinction between double and single sceptics has important implications for climate policy, efforts to reduce emissions and public engagement. While previous research shows that it is difficult to erode sceptical attitudes that are psychologically motivated [9, 12, 21, 34], our findings suggest that efforts to build trust in scientists, public education campaigns and targeted economic support could help erode scepticism (at least among single-issue sceptics) and increase compliance with behavioral remedies to mitigate climate change and the pandemic among most climate sceptics and antivaxxers.

Our data facilitate comparisons across eight countries critical for global climate and COVID-19 mitigation. However, geographical diversity does create challenges. For example, it was not possible to gather data on all factors shown to impact scepticism towards climate change and vaccination in previous research. In particular, political sensitivities did not allow asking about ideological orientation in China [60] while the well-known difficulty of eliciting accurate disclosure of income from survey responses [61] led to these variables being excluded from our analysis. Furthermore, whilst enlisting a reputable survey firm facilitated the acquisition of comparable public opinion data from eight key diverse countries, our reliance on urban recruits from rapidly growing economies highlights a need for more direct fieldwork with rural populations in industrialising contexts to facilitate better understandings of sceptical attitudes among these important groups for policymakers. We also acknowledge that causality might flow in the opposite direction whereby scepticism on climate change and COVID-19 vaccination increase distrust in scientists or, as others have previously suggested, scepticism on climate change [35] (and vaccines) and trust in scientists interactively influence each other.

Moreover, since climate sceptics and antivaxxers only comprise a small share of national populations, attempts to elucidate the drivers behind hard-core sceptics drawing on large-N samples are ultimately based on a relatively small number of respondents. Importantly, this raises potential questions about the sample size, power and sensitivity of analysis. Yet *post hoc* assessments of our core regressions in Table 3 are overwhelmingly promising (S21 Table in S1 File) and suggest that the power of all pooled and most national regressions was above 0.9. Repeating our base models with wider configurations of sceptic profiles that span weaker sceptic attitudes towards climate change and COVID-19 vaccination (namely: the belief that climate change is a minor threat and being very unlikely to take a COVID-19 vaccine) suggest that our findings are robust to power sensitivity concerns. Consistent with our main results from Table 3, the difference in the (absolute) size of the trust estimates across the wider configurations (S22 Table in S1 File) suggest that trust in scientists is a significantly (two-to-three-fold) stronger predictor of double, as opposed to single-issue, sceptics. As wider configurations with larger numbers of sceptic profiles yield similar results, our post-hoc sensitivity analyses suggest that the variation in the predictive power of trust across different sceptic profiles is unlikely to be (at least entirely) attributable to the small numbers of positive occurrences. Indeed, in some sense, our restrictive sceptic profiling constitutes a harder test for assessing the role of trust in scientists which uncovers robust findings despite the relatively small number of sceptics sampled.

Moreover, even within this select group, we find significant diversity in socio-demographic characteristics, political orientation, and levels of climate knowledge. Although this suggests scepticism may be linked to several different variables, it also implies that attempts to dispel scepticism will need fine-tuning to target different sceptic profiles (and sources). Nyhan et al.’s work on the ‘backfire effect’, for example, suggests that corrective scientific communication would reduce misperceptions for most science sceptics, but reinforce them for those whose scepticism is motivated by a need to defend their underlying (sceptic) worldviews [62]. For the latter, technique rebuttal—encouraging sceptics to build trust in scientists by engaging in respectful exchanges about scientific perceptions—is likely to be more effective [63, 64]. Similarly, corrective strategies are also likely to vary in their ability to dispel scepticism across different issues depending on the source(s) of science denial [65]. Therefore, further efforts to target sceptics are needed to improve understandings about these relatively diverse small groups.

## Conclusion

We analysed the associations between distrust in scientists and scepticism towards climate change and COVID vaccination at the peak of the pandemic in 2021. We found that for most sceptics, who are sceptical towards either climate change or COVID vaccination (but not both), scepticism is strongly linked to a distrust in scientists. However, for an important minority of hardcore sceptics who are sceptical towards both domains, scepticism appears to be motivated by an underlying sceptical mindset rather than a narrower distrust in scientists. A key implication of this research is that, when trying to dispel scepticism towards societal responses to major national and global problems, policymakers should pursue a tailored approach that accounts for different sceptic profiles. For most sceptics—who we call ‘single-issue’ sceptics—efforts to overcome relatively isolated predictors of scepticism such as building trust in scientists, information campaigns and economic support are likely to increase support for policies entailing societal responses to mitigate global challenges such as climate change and pandemics. By contrast, such strategies are likely to be ineffective or even counter-productive for an important minority of hardcore sceptics whose scepticism is associated with a more generalised sceptical worldview.

## Supporting information

S1 File Supporting information. (DOCX)
