# Supplementary material for: How to distinguish climate sceptics, antivaxxers, and persistent sceptics: Evidence from a multi-country survey of public attitudes
Source: PLoS One. 2024 Oct 2;19(10):e0310325. doi: 10.1371/journal.pone.0310325 (PMC11446448; doi:10.1371/journal.pone.0310325)
Supplement: S1 File — (DOCX) [file pone.0310325.s001.docx]

**How to distinguish climate sceptics, antivaxxers, and perpetual sceptics Supplementary Information**

**Supplementary Table S1: Pairwise correlation matrix of trust in university scientists and other elite social institutions**

| Sample | Trust in national government | Trust in television news |
| --- | --- | --- |
| Pooled | 0.28*** | 0.40*** |
| Australia | 0.19*** | 0.30*** |
| Brazil | -0.09*** | 0.36*** |
| China | X | 0.40*** |
| UK | 0.25*** | 0.39*** |
| India | 0.42*** | 0.37*** |
| Japan | 0.36*** | 0.47*** |
| South Africa | 0.33*** | 0.41*** |
| US | 0.43*** | 0.51*** |

Note: Entries are Pearson’s correlation coefficients for trust in university scientists, TV news and national government for providing accurate information on sustainable energy and environmental issues. X denotes missing as respondents were not asked to rate trust in national government in China. *** denotes p<0.001, ** p<0.01 and * p<0.05.

**Supplementary Table S2: Variables and coding strategy**

| **Variable** | **Description** | **Coding/ scale** | **Number of missing values (maximum total=16,035)** |
| --- | --- | --- | --- |
| Age | Age in years | Range (18-99) | 0 |
| Gender | Binary variable | 0=male, 1=female | 0 |
| Degree | Binary variable | 0=no bachelor degree, 1= bachelor degree | 0 |
| EnvTop3 | Rates the environment as a top-three priority | *Which of the following, if any, do you think are the most important issues facing your country at this time: health, immigration, crime, the economy, tax, pensions, education, family life & childcare, international relations, the environment and transport. (Select up to 3 answers)*  0=does not rate environment as top-three priority, 1=rates environment as top-three priority | *0* |
| HealthTop3 | Rates health as a top-three priority | *Which of the following, if any, do you think are the most important issues facing your country at this time: health, immigration, crime, the economy, tax, pensions, education, family life & childcare, international relations, the environment and transport. (Select up to 3 answers)*  0=does not rate health as top-three priority, 1=rates health as top-three priority | *0* |
| Objective knowledge | Number of correct responses to energy, climate and virus knowledge questions | 0 (0 correct science knowledge answers) to 9 (9 correct science knowledge answers) | 0 |
| Self-declared knowledge | Self-declared knowledge about energy production, delivery and usage | *How knowledgeable, if at all, would you say you are about how energy is produced, delivered and used?*  1 (not at all knowledgeable) to 5 (very knowledgeable) | *0* |
| Trust in scientists | Level of trust in university scientists for accurate information on sustainable energy and environmental issues | *To what extent, if at all, do you trust the following sources to provide you with accurate information on sustainable energy and environmental issues? Seven-point scale:*  1 (do not trust at all) to 7 (trust completely) | *475* |
| Perceived income sufficiency | Perceived income sufficiency | *Which of the following best describes how you feel about your household’s income nowadays?* 1=living comfortably on present income, 2=coping on present income, 3=finding it difficult on present income, 4=finding it very difficult on present income, 5=prefer not to say, 6=don’t know | *357* |
| ClimateResp | Attribution of responsibility to the individual versus other institutions for combatting climate change | *How much responsibility, if any, do you think individuals have solving climate change compared to other institutions (e.g. governments, businesses, charities)? 11-point scale:*  From 0 (completely the responsibility of other institutions) to 10 (completely the individual’s responsibility to solve) | *0* |
| CovidResp | Attribution of responsibility to the individual versus other institutions for combatting the COVID-19 pandemic | *How much responsibility, if any, do you think individuals have solving COVID-19 compared to other institutions (e.g. governments, businesses, charities)? 11-point scale:*  From 0 (completely the responsibility of other institutions) to 10 (completely the individual’s responsibility to solve) | *0* |
| Precautionism | Preference for taking immediate action to prevent a potentially serious societal problem versus waiting for more certain information | In general, do you think it is better to: take action in anticipation of what may become a serious problem based on uncertain information or wait to see if the problem develops into a serious problem and take action then? 11-point scale:  From 0 (definitely take action immediately) to 10 (definitely wait and see) | 366 |
| Climate sceptic | Feels that climate change does not pose any threat to their country (and not an antivaxxer) | Do you think that climate change is a major threat, a minor threat or not a threat to your country?  0 = not a climate sceptic (a major or minor threat)  1 = climate sceptic (not a threat) | 0 |
| Antivaxxer | Would definitely not take a COVID-19 vaccine if offered one (and not a climate sceptic) | *It was recently announced that effective COVID-19 vaccines have been developed. If a COVID-19 vaccine is offered to you, how likely or unlikely are you take it?*  0 = not an antivaxxer (any of the following responses: Already taken it, would definitely take it, somewhat likely to take it, neither likely not unlikely to take it, somewhat unlikely to take it, and very unlikely to take it)  1= antivaxxer (would definitely not take it) | *0* |
| Double sceptic | Feels that climate change does not pose any threat to their country and would definitely not take a COVID-19 vaccine if offered one | 0 = not a double sceptic  1 = double sceptic | 0 |
| EconOver  Clim&Covid | Gives complete priority to economic growth over protecting the climate and public from COVID-19 | 0 = does not give complete priority to economic growth over protecting the climate and public from COVID-19  1 = gives complete priority to economic growth over protecting the climate and public from COVID-19 (i.e. if respondent gave ‘10’ points to the economy over climate change and COVID-19 as coded in EconOverClim/Covid variables below. | 0 |
| EconOver  Clim | Gives complete priority to economic growth over protecting climate change (and does not give complete priority to economy over protecting the public from COVID-19) | *Some believe that economic growth should have priority even if that hinders protecting the climate. Others believe that protecting the climate should have priority even if that reduces economic growth. On a scale of 1 to 10, where would you place yourself, where 0 means economic growth should have priority and 10 means protecting the climate should have priority*  0 = economic growth should not have complete priority  10 = economic growth should have complete priority. Coded as 0 if response was 0 to 9, coded as 1 if response was equal to 10. | *0* |
| EconOver  Covid | Gives complete priority to economic growth over protecting the public from COVID-19 (and does not give complete priority to economy over protecting the climate) | *Some believe that economic growth should have priority even if that hinders protecting the public from COVID-19. Others believe that protecting the public from COVID-19 should have priority even if that reduces economic growth. On a scale of 1 to 10, where would you place yourself, where 0 means economic growth should have priority and 10 means protecting the public from COVID-19 should have priority*  0 = economic growth should not have complete priority  10 = economic growth should have complete priority. Coded as 0 if response was 0 to 9, coded as 1 if response was equal to 10. | *0* |
| Political orientation | Self-placement on 11-point Likert scale representing the political left-to-right. | The expressions “left” and “right” are often used to describe a person’s political position. Where would you place yourself on this scale? 1=left to 10=right, 11=don’t know, 12= prefer not to say | *5428* |

| *Discrete variables* | | |
| --- | --- | --- |
| *Variable* | *N* | *% share* |
| Double sceptic | 34 | 1.70 |
| Climate sceptic | 155 | 7.75 |
| Antivaxxer | 88 | 4.40 |
| EconOverClim&Covid | 30 | 1.50 |
| EconOverClim | 119 | 5.95 |
| EconOverCovid | 26 | 1.30 |
| Gender |  |  |
| *Female* | 1020 | 51.00 |
| *Male* | 980 | 49.00 |
| Educational attainment |  |  |
| *No completed education* | 7 | 0.35 |
| *Primary school only* | 23 | 1.15 |
| *Secondary education to Year 11 or below* | 229 | 11.45 |
| *Secondary education to Year 12* | 313 | 15.65 |
| *TAFE Certificate, Diploma* | 598 | 29.90 |
| *University and tertiary education (Undergraduate, Bachelor degrees)* | 567 | 28.35 |
| *Masters degree* | 211 | 10.55 |
| *Doctorate/PhD* | 38 | 1.90 |
| *Prefer not to answer* | 14 | 0.70 |
| EnvTop3 | 801 | 40.05 |
| HealthTop3 | 1144 | 57.20 |
| *Continuous variables* | | |
| *Variable* | *Mean* | *SD* |
| Age | 46.98 | 17.42 |
| Know. Index | 3.94 | 1.81 |
| Energy know. (self-decl.) | 2.88 | 1.05 |
| Trust scientists | 4.97 | 1.44 |
| Perceived income sufficiency | 2.08 | 0.98 |
| Climate resp. | 5.87 | 2.17 |
| Covid resp. | 5.89 | 2.26 |
| Precautionism | 6.58 | 2.55 |

**Suppleme**n**tary Tables S3-S10: Descriptive Statistics for Country Samples**

Supplementary Table S3: Descriptive statistics for Australia (n=2000)

| *Discrete variables* | | |
| --- | --- | --- |
| *Variable* | *N* | *% share* |
| Double sceptic | 23 | 1.15 |
| Climate sceptic | 73 | 3.65 |
| Antivaxxer | 72 | 3.60 |
| EconOverClim&Covid | 67 | 3.35 |
| EconOverClim | 150 | 7.50 |
| EconOverCovid | 62 | 3.10 |
| Gender |  |  |
| *Female* | 1040 | 52.00 |
| *Male* | 960 | 48.00 |
| Educational attainment |  |  |
| *Without formal education* | 4 | 0.20 |
| *Elementary education* | 30 | 1.50 |
| *Lower secondary education* | 26 | 1.30 |
| *Complete lower secondary education* | 55 | 2.75 |
| *Upper secondary education* | 715 | 35.75 |
| *University* | 794 | 39.70 |
| *Postgraduate degree* | 272 | 13.60 |
| *Master’s degree* | 68 | 3.40 |
| *Doctor’s degree* | 26 | 1.30 |
| *Prefer not to answer* | 10 | 0.50 |
| EnvTop3 | 414 | 20.70 |
| HealthTop3 | 1645 | 82.25 |
| *Continuous variables* | | |
| *Variable* | *Mean* | *SD* |
| Age | 42.82 | 15.67 |
| Know. Index | 2.80 | 1.61 |
| Energy know. (self-decl.) | 3.21 | 1.17 |
| Trust scientists | 5.12 | 1.65 |
| Perceived income sufficiency | 2.11 | .97 |
| Climate resp. | 5.87 | 2.67 |
| Covid resp. | 5.92 | 2.82 |
| Precautionism | 7.91 | 2.93 |

Supplementary Table S4: Descriptive statistics for Brazil (n=2000)

| *Discrete variables* | | |
| --- | --- | --- |
| *Variable* | *N* | *% share* |
| Double sceptic | 2 | 0.10 |
| Climate sceptic | 82 | 4.10 |
| Antivaxxer | 10 | 0.50 |
| EconOverClim&Covid | 11 | 0.55 |
| EconOverClim | 37 | 1.85 |
| EconOverCovid | 19 | 0.95 |
| Gender |  |  |
| *Female* | 997 | 49.85 |
| *Male* | 1003 | 51.15 |
| Educational attainment |  |  |
| *No formal schooling* | 4 | 0.20 |
| *Primary education incomplete* | 2 | 0.10 |
| *Primary education complete* | 14 | 0.70 |
| *Junior high school incomplete* | 8 | 0.40 |
| *Junior high school complete* | 31 | 1.55 |
| *Junior/ technical secondary school* | 32 | 1.60 |
| *Senior/ technical secondary school* | 81 | 4.05 |
| *Senior high school* | 123 | 6.15 |
| *College* | 352 | 17.60 |
| *University* | 1222 | 61.20 |
| *Postgraduate* | 129 | 6.45 |
| *Prefer not to say* | 2 | 0.10 |
| EnvTop3 | 995 | 49.75 |
| HealthTop3 | 1042 | 52.10 |
| *Continuous variables* | | |
| *Variable* | *Mean* | *SD* |
| Age | 41.53 | 15.42 |
| Know. Index | 4.62 | 1.40 |
| Energy know. (self-decl.) | 3.21 | 0.94 |
| Trust scientists | 5.13 | 1.27 |
| Perceived income sufficiency | 1.54 | 0.66 |
| Climate resp. | 6.15 | 2.09 |
| Covid resp. | 6.10 | 2.31 |
| Precautionism | 6.58 | 2.52 |

Supplementary Table S5: Descriptive statistics for China (n=2000)

| *Discrete variables* | | |
| --- | --- | --- |
| *Variable* | *N* | *% share* |
| Double sceptic | 11 | 0.55 |
| Climate sceptic | 79 | 3.95 |
| Antivaxxer | 51 | 2.55 |
| EconOverClim&Covid | 24 | 1.20 |
| EconOverClim | 82 | 4.10 |
| EconOverCovid | 20 | 1.00 |
| Gender |  |  |
| *Female* | 1023 | 51.15 |
| *Male* | 977 | 48.85 |
| Educational attainment |  |  |
| *Primary education* | 11 | 0.55 |
| *Lower secondary education* | 336 | 16.80 |
| *Upper secondary education* | 409 | 20.45 |
| *Post-secondary education, but not university* | 367 | 18.35 |
| *First degree* | 540 | 27.00 |
| *Postgraduate degree* | 324 | 16.20 |
| *Prefer not to answer* | 13 | 0.65 |
| EnvTop3 | 646 | 32.30 |
| HealthTop3 | 1523 | 76.15 |
| *Continuous variables* | | |
| *Variable* | *Mean* | *SD* |
| Age | 47.83 | 16.87 |
| Know. Index | 4.23 | 1.77 |
| Energy know. (self-decl.) | 2.77 | 1.03 |
| Trust scientists | 5.12 | 1.33 |
| Perceived income sufficiency | 1.96 | 0.89 |
| Climate resp. | 5.71 | 2.02 |
| Covid resp. | 5.63 | 2.26 |
| Precautionism | 6.71 | 2.43 |

Supplementary Table S6: Descriptive statistics for the UK (n=2000)

| *Discrete variables* | | |
| --- | --- | --- |
| *Variable* | *N* | *% share* |
| Double sceptic | 4 | 0.20 |
| Climate sceptic | 73 | 3.65 |
| Antivaxxer | 75 | 3.75 |
| EconOverClim&Covid | 146 | 7.30 |
| EconOverClim | 142 | 7.10 |
| EconOverCovid | 67 | 3.35 |
| Gender |  |  |
| *Female* | 981 | 49.05 |
| *Male* | 1019 | 50.95 |
| Educational attainment |  |  |
| *No formal schooling* | 6 | 0.30 |
| *School up to 2^nd^ grade* | 6 | 0.30 |
| *School up to 4^th^ grade* | 9 | 0.45 |
| *School up to 9^th^ grade* | 13 | 0.65 |
| *SSC (10^th^ grade)* | 59 | 2.95 |
| *HSC (12^th^ grade)* | 152 | 7.60 |
| *Some college* | 176 | 8.80 |
| *Graduate/ Postgraduate – General* | 882 | 44.10 |
| *Graduate/ Postgraduate - Professional* | 686 | 34.30 |
| *Prefer not to answer* | 11 | 0.55 |
| EnvTop3 | 670 | 33.50 |
| HealthTop3 | 1103 | 55.15 |
| *Continuous variables* | | |
| *Variable* | *Mean* | *SD* |
| Age | 38.61 | 14.88 |
| Know. Index | 4.11 | 1.34 |
| Energy know. (self-decl.) | 3.86 | 1.10 |
| Trust scientists | 5.49 | 1.50 |
| Perceived income sufficiency | 2.16 | 1.21 |
| Climate resp. | 4.98 | 3.02 |
| Covid resp. | 4.82 | 3.08 |
| Precautionism | 6.51 | 3.49 |

Supplementary Table S7: Descriptive statistics for India (n=2000)

| *Discrete variables* | | |
| --- | --- | --- |
| *Variable* | *N* | *% share* |
| Double sceptic | 12 | 0.60 |
| Climate sceptic | 85 | 4.18 |
| Antivaxxer | 51 | 2.52 |
| EconOverClim&Covid | 20 | 0.98 |
| EconOverClim | 41 | 2.01 |
| EconOverCovid | 16 | 0.79 |
| Gender |  |  |
| *Female* | 1054 | 51.79 |
| *Male* | 981 | 48.21 |
| Educational attainment |  |  |
| *Primary education/ junior high school* | 72 | 3.54 |
| *High school* | 643 | 31.60 |
| *Post-secondary education* | 419 | 20.59 |
| *First degree* | 801 | 39.36 |
| *Postgraduate degree* | 70 | 3.44 |
| *Doctorate or other advanced degree* | 16 | 0.79 |
| *Prefer not to answer* | 14 | 0.69 |
| EnvTop3 | 370 | 18.18 |
| HealthTop3 | 972 | 47.76 |
| *Continuous variables* | | |
| *Variable* | *Mean* | *SD* |
| Age | 50.28 | 16.97 |
| Know. Index | 3.98 | 1.81 |
| Energy know. (self-decl.) | 2.69 | 0.98 |
| Trust scientists | 4.29 | 1.26 |
| Perceived income sufficiency | 2.41 | 0.98 |
| Climate resp. | 6.27 | 1.96 |
| Covid resp. | 5.99 | 2.20 |
| Precautionism | 4.78 | 2.34 |

Supplementary Table S8: Descriptive statistics for Japan (n=2035)

| *Discrete variables* | | |
| --- | --- | --- |
| *Variable* | *N* | *% share* |
| Double sceptic | 37 | 1.85 |
| Climate sceptic | 44 | 2.20 |
| Antivaxxer | 346 | 17.30 |
| EconOverClim&Covid | 89 | 4.45 |
| EconOverClim | 170 | 8.50 |
| EconOverCovid | 127 | 6.35 |
| Gender |  |  |
| *Female* | 1028 | 51.40 |
| *Male* | 972 | 48.60 |
| Educational attainment |  |  |
| *No schooling* | 1 | 0.05 |
| *Some primary* | 2 | 0.10 |
| *Completed primary* | 6 | 0.30 |
| *Secondary (grades 8-9)* | 31 | 1.55 |
| *Secondary (grades 10-11)* | 90 | 4.50 |
| *Secondary (matric or equivalent)* | 649 | 32.45 |
| *Post-secondary education, but not university* | 568 | 28.40 |
| *First degree* | 461 | 23.05 |
| *Postgraduate degree* | 178 | 8.90 |
| *Prefer not to answer* | 14 | 8.90 |
| EnvTop3 | 174 | 8.70 |
| HealthTop3 | 1275 | 63.75 |
| *Continuous variables* | | |
| *Variable* | *Mean* | *SD* |
| Age | 39.19 | 14.87 |
| Know. Index | 4.22 | 1.46 |
| Energy know. (self-decl.) | 3.22 | 1.10 |
| Trust scientists | 5.18 | 1.51 |
| Perceived income sufficiency | 2.76 | 1.01 |
| Climate resp. | 5.36 | 2.58 |
| Covid resp. | 4.85 | 2.82 |
| Precautionism | 7.12 | 3.05 |

Supplementary Table S9: Descriptive statistics for South Africa (n=2000)

| *Discrete variables* | | |
| --- | --- | --- |
| *Variable* | *N* | *% share* |
| Double sceptic | 80 | 4.00 |
| Climate sceptic | 205 | 10.25 |
| Antivaxxer | 123 | 6.15 |
| EconOverClim&Covid | 82 | 4.10 |
| EconOverClim | 126 | 6.30 |
| EconOverCovid | 51 | 2.55 |
| Gender |  |  |
| *Female* | 974 | 48.70 |
| *Male* | 1026 | 51.30 |
| Educational attainment |  |  |
| *Some high school or less* | 42 | 2.10 |
| *High school degree or equivalent* | 319 | 15.95 |
| *Some college – no degree* | 440 | 22.00 |
| *2-year college/ technical degree* | 240 | 12.00 |
| *4-year college degree* | 624 | 31.20 |
| *Postgraduate degree* | 332 | 16.60 |
| *Prefer not to answer* | 3 | 0.15 |
| EnvTop3 | 594 | 29.70 |
| HealthTop3 | 1209 | 60.45 |
| *Continuous variables* | | |
| *Variable* | *Mean* | *SD* |
| Age | 47.91 | 17.44 |
| Know. Index | 3.91 | 1.69 |
| Energy know. (self-decl.) | 2.99 | 1.07 |
| Trust scientists | 4.75 | 1.59 |
| Perceived income sufficiency | 1.96 | 1.04 |
| Climate resp. | 5.67 | 2.28 |
| Covid resp. | 5.68 | 2.44 |
| Precautionism | 6.56 | 2.52 |

Supplementary Table S10: Descriptive statistics for the US (n=2000)

**Supplementary Table S11: Pooled model results showing effect of key drivers on the relative log odds of being a double sceptic, climate sceptic or antivaxxer**

| Parameter | Model | | |
| --- | --- | --- | --- |
|  | 1A | 2A | 3A |
|  | Climate sceptic and antivaxxer | Climate sceptic only | Antivaxxer only |
| Age | 0.03 | 0.02** | -0.01T |
| Female | -0.23 | -0.67T | 0.19* |
| Degree | -0.40* | -0.09T | -0.33*** |
| Prioritise environment | -1.66*** | -1.44 | -0.16 |
| Prioritise health | -1.10*** | -0.58T | -0.22** |
| Objective knowledge | -0.03 | -0.16*** | -0.01 |
| Self-declared energy knowledge | 0.15T | 0.12** | 0.01 |
| Trust in scientists | -0.56*** | -0.32** | -0.25*** |
| Perceived income sufficiency | 0.04 | -0.04 | 0.25*** |
| Climate responsibility | -0.01 | -0.12*** | 0.04* |
| COVID-19 responsibility | -0.16*** | 0.04* | -0.01* |
| Precautionism | 0.05 | -0.06*** | 0.04** |
| N | 203 | 796 | 816 |
| R2 | 0.27 | 0.16 | 0.14 |

Table S11: Effects on the relative log odds of being a climate sceptic, antivaxxer or both (N=14956).

Note: The dependent variable is binary, taking the value of 1 if an individual response is categorized as the defined sceptic attitude towards climate change and COVID-19 and 0 otherwise. Model (1A) estimates the probability of an individual being both climate sceptic and antivaxxer, model (2A) climate sceptic but not antivaxxer and model (3A) antivaxxer but not climate sceptic. Country controls are included but not reported. Individual country regressions are reported in Table 3. T denotes P<0.10, *P<0.05, **P<0.01 and ***P<0.001.

**Supplementary Table S12: Pooled model results showing effect of key drivers on the relative log odds of giving complete priority to the economy over climate protection or combatting COVID-19 or both**

| Parameter | Model | | |
| --- | --- | --- | --- |
|  | 1B | 2B | 3B |
|  | Prioritise economy over both climate and COVID-19 | Prioritise economy over climate only | Prioritise economy over COVID-19 only |
| Age | 0.01* | 0.01*** | 7.09E-4 |
| Female | -0.03 | -0.07 | -0.04 |
| Degree | 0.12 | -0.23** | -0.23T |
| Prioritise environment | -0.87*** | -0.90*** | -0.16 |
| Prioritise health | -0.66*** | -0.22** | -0.58*** |
| Objective knowledge | -0.15*** | -0.20*** | 0.02 |
| Self-declared energy knowledge | 0.57*** | 0.23*** | 0.13** |
| Trust in scientists | -0.21*** | -0.11*** | -0.15*** |
| Economic hardship | 0.04 | 0.11** | 0.11* |
| Climate responsibility | -0.12*** | -0.01 | -0.02 |
| COVID-19 responsibility | -0.18*** | 0.03T | -0.13*** |
| Precautionism | 0.23*** | 0.11*** | 0.08*** |
| N | 469 | 867 | 388 |
| R2 | 0.23 | 0.08 | 0.10 |

Table S12: Effects on the relative log odds of giving complete priority to the economy over climate protection or combatting COVID-19 or both (N=14956).

Note: The dependent variable is binary, taking the value of 1 if an individual response is categorized as the defined sceptic attitude towards climate change and COVID-19 and 0 otherwise. Model (1B) estimates the probability of an individual giving complete priority to the economy over combatting climate change and the pandemic, model (2B) the probability of giving complete priority to the economy over climate protection but not combatting COVID-19 and model (3B) the probability of giving complete priority to the economy over combatting COVID-19 but not climate protection. Country controls are included but not reported. Individual country regressions are reported in Table 4. T denotes P<0.10, *P<0.05, **P<0.01 and ***P<0.001.

In comparing tables S11 and S12 (equivalent to models A and B in Table 1 the main text), it is noteworthy that the numbers of climate sceptics and those who deprioritise climate is relatively comparable whereas more than twice as many respondents prioritise the economy over both climate and COVID-19 compared to the number that are both anti-vaxxers and climate sceptics.

**Supplementary Table S13: Probability of making no lifestyle changes due to climate change or COVID-19**

| Trust in scientists | Model | | |
| --- | --- | --- | --- |
|  | 1B | 2B | 3B |
|  | No lifestyle changes due to climate change or COVID-19 | No lifestyle changes due to climate change only | No lifestyle changes due to COVID-19 only |
| 1 | 0.07*** | 0.16*** | 0.02*** |
| 2 | 0.05*** | 0.15*** | 0.02*** |
| 3 | 0.03*** | 0.13*** | 0.02*** |
| 4 | 0.03*** | 0.12*** | 0.02*** |
| 5 | 0.02*** | 0.11*** | 0.02*** |
| 6 | 0.02*** | 0.09*** | 0.01*** |
| 7 | 0.01*** | 0.08*** | 0.01*** |

Table S13: Predicted probability of making no lifestyle changes due to COVID-19, climate change or both issues depending on level of trust in scientists.

Note: The dependent variable is binary, taking the value of 1 if an individual reports making no lifestyle changes due to the specified issue combination and 0 otherwise. Model (1) estimates the probability of an individual reporting no lifestyle changes due to climate change and the pandemic, model (2) the probability of no lifestyle changes due to climate change but not COVID-19 and model (3) the probability of no lifestyle changes due to COVID-19 but not climate change. Country controls are included but not reported. Individual country regressions are reported in Table 4. *P<0.05, **P<0.01 and ***P<0.001.

**Secondary predictor estimates in Table 2**

While estimates obtained for most other predictor variables are relatively smaller in (absolute) magnitude compared to the trust in scientist coefficients discussed in the main text, several obtained statistical significance, suggesting that they are important, albeit, secondary sources of scepticism towards climate change and COVID vaccination. Specifically; the positive significant (P<0.001) age estimate in models 2B and 2B consistently suggest that older people are more likely to be climate sceptics and prioritise the economy over climate change without being sceptical towards COVID vaccination or prioritizing the economy over COVID mitigation. However, the small size of the estimates suggest that the association is relatively weak: being ten years older makes one less than 0.01 percent more likely to be climate sceptics or economy-over-climate prioritisers. The negative and positive signs of the female estimates in Models 2A and 3A respectively indicate that women are less likely to be climate sceptics (without being antivaxxers) , but more likely to be antivaxxers (without being climate sceptics) compared to men. However, gender was not associated with economy priotisers in Model B. Possession of a university degree was inversely associated with double sceptics (Model A1), antivaxxers (Model A3), economy-over-climate (Model 2B) and economy-over-COVID (Model 3C) prioritisers: with degree holders one percent less likely to hold each of the latter three sceptic profiles compared to non-degree holders. As expected and elaborated in the main text, people who rated health or the environment as a top priority were significantly – one to six percent (P<0.001) - less likely to hold hardcore sceptic profiles or be climate sceptics (or prioritise the economy over the climate), although no comparable association was found for antivaxxer or economy-over-COVID only profiles. Objective knowledge was negatively associated with climate sceptic (and equivalent economy-over-climate) only profiles: for each correct answer to the nine science knowledge questions, respondents were one percent less likely to climate sceptics or economy-over-climate prioritisers. Yet self-assessed energy knowledge was, by contrast, associated with higher probability of holding (climate) sceptic or economy-prioritising positions: a one-point increase in respondents’ zero to five point self-rating of energy knowledge was associated with a one percent increase in the likelihood of being a climate sceptic (Model A2) , prioritising the economy over both the climate and COVID (Model B1) or climate (B2) or COVID (B3) separately . A one-point increase in the five-point perceived economic hardship scale was associated with a percent increase in the likelihood of being an antivaxxer (Model A3) and economy over climate prioritiser (Model B2). While perceived responsibility for combatting climate change or COVID-19 tended to be associated with reduced probabilities of all sceptic and economy-prioritiser profiles, the estimated effect sizes were negligible. By contrast, even a small increase in precautionary individuals were significantly (P<0.001) more likely to prioritise the economy over climate and COVID (Model B1), climate (Model B2) and COVID-19 (Model B3) only; with a one-point increase in the 11-point self-rated precautionary scale associated with a one percent increase in probability of holding the former two economy-prioritiser profiles.

**Supplementary Tables S14-S17: Comparing political ideology, distrust in scientists, government and television news and associated correlations across non-sceptic and sceptic segments**

| **Variable** | **Non-sceptic (n=14423)** | | **Double sceptic (n=203)** | | **Climate sceptic (n=796)** | | **Antivaxxer (n=816)** | |
| --- | --- | --- | --- | --- | --- | --- | --- | --- |
|  | **Mean / n** | **SD/ % share** | **Mean / n** | **SD/ % share** | **Mean/ n** | **SD/ % share** | **Mean/ n** | **SD/ % share** |
| Left-right orientation | 5.44 | 0.03 | 7.45 | 0.21 | 7.06 | 0.11 | 5.98 | 0.13 |
| Distrust scientists | 1.90 | 0.01 | 3.94 | 0.13 | 3.07 | 0.06 | 2.56 | 0.06 |
| Distrust government | 3.21 | 0.02 | 4.41 | 0.14 | 3.48 | 0.07 | 4.06 | 0.07 |
| Distrust TV news | 2.84 | 0.01 | 5.07 | 0.11 | 3.76 | 0.07 | 3.74 | 0.07 |
| Far-left | 529 | 3.72 | 5 | 2.46 | 15 | 1.88 | 50 | 6.13 |
| Far-right | 991 | 6.97 | 61 | 30.05 | 133 | 16.71 | 117 | 14.34 |

Table S14: Descriptive statistics of key variables across sceptic and non-sceptic segments.

| **Variable** | **Double vs. single sceptics** | | | | **Non-sceptics vs. sceptics** | | | | | |
| --- | --- | --- | --- | --- | --- | --- | --- | --- | --- | --- |
|  | **Double sceptic vs. climate sceptic** | | **Double sceptic vs. antivaxxer** | | **Non-sceptic vs. double sceptic** | | **Non-sceptic vs. climate sceptic** | | **Non-sceptic vs. antivaxxer** | |
|  | **t-test statistic** | **p-value** | **t-test statistic** | **p-value** | **t-test statistic** | **p-value** | **t-test statistic** | **p-value** | **t-test statistic** | **p-value** |
| Left-right orientation | 36.39 | 0.000 | 125.50 | 0.000 | -7.4E2 | 0.000 | -1.20E3 | 0.000 | -3.60E2 | 0.000 |
| Distrust scientists | 139.46 | 0.000 | 222.71 | 0.000 | -1.6E3 | 0.000 | -1.80E3 | 0.000 | -1.10E3 | 0.000 |
| Distrust government | 133.26 | 0.000 | 50.46 | 0.000 | -6.6E2 | 0.000 | -2.90E2 | 0.000 | -7.10E2 | 0.000 |
| Distrust TV news | 208.93 | 0.000 | 213.13 | 0.000 | -1.9E3 | 0.000 | -1.30E3 | 0.000 | -1.30E3 | 0.000 |
| Far-left | 0.53 | 0.598 | -2.07 | 0.038 | 0.93 | 0.351 | 2.68 | 0.007 | -3.48 | 0.001 |
| Far-right | 4.28 | 0.000 | 5.41 | 0.000 | -12.53 | 0.000 | -10.16 | 0.000 | -17.85 | 0.000 |

Table S15: Comparing left-right orientation, distrust in scientists, government and television news across non-sceptic and sceptic segments.

Note: (Unequal) t-test statistics and associated p-values indicate that the difference in means/ percentage share (of respondents possessing far-left or far-right political orientation) is statistically significant at the 0.001 level in all cases.

| **Sample** | **Double sceptic vs. climate sceptic** | | **Double sceptic vs. antivaxxer** | |
| --- | --- | --- | --- | --- |
|  | **t-test statistic** | **p-value** | **t-test statistic** | **p-value** |
| Pooled | 1.79 | 0.037 | 0.89 | 0.188 |
| Australia | 0.11 | 0.456 | -0.44 | 0.33 |
| Brazil | -0.24 | 0.405 | -0.12 | 0.452 |
| China | X |  | X |  |
| UK | 0.36 | 0.359 | -0.03 | 0.488 |
| India | 0.19 | 0.427 | 0.11 | 0.456 |
| Japan | -0.20 | 0.421 | 0 | 0.500 |
| South Africa | -0.09 | 0.461 | 0.17 | 0.432 |
| US | -1.38 | 0.083 | -0.99 | 0.16 |

Table S16: Significance of the difference between correlation coefficients between trust in scientists, double sceptics, climate sceptics and antivaxxers.

Note: X = Sample size too small to compare coefficients.

| Sample | Double sceptic | Climate sceptic | Antivaxxer |
| --- | --- | --- | --- |
| Pooled | 203 | 796 | 816 |
| Australia | 34 | 155 | 88 |
| Brazil | 23 | 73 | 72 |
| China | 2 | 82 | 10 |
| UK | 11 | 79 | 51 |
| India | 4 | 73 | 75 |
| Japan | 12 | 85 | 51 |
| South Africa | 37 | 44 | 346 |
| US | 80 | 205 | 123 |

Table S17: Number of respondents that fitted the sceptic profiles in the pooled and country samples.

**Supplementary Figures S1-S2: Political orientation, trust in university scientists, national government and television news across countries and national sceptic segments**

Supplementary Figure S1: Mean left-right orientation and distrust in university scientists, national government and television news across countries. Blue bars show the mean score respondents assigned when asked to locate themselves on a left-right political spectrum ranging from 0 (left) to 10 (right). Orange lines show mean levels of respondents’ distrust in university scientists, grey lines their national government and yellow lines television news from 0 (completely trust) to 6 (do not trust at all). Chinese respondents were not asked questions on left-right orientation and distrust in national government.

Supplementary Figure S2: Mean values of four key variables across non-sceptic, double sceptic and single sceptic segments in separate country samples. The blue bars (left) show the mean score respondents assigned when asked to locate themselves on a left-right political scale from 0 (left) to 10 (right). Brown bars (second) show mean levels of respondents’ distrust in university scientists, green bars (third) their national government and orange bars (fourth) television news from 0 (completely trust) to 6 (do not trust at all).

**Supplementary Tables S18-S19: Associations with climate sceptics, antivaxxers, double sceptics and associated prioritisations of the economy across countries**

| Country | Age | | Female | Prioritise env | Prioritise health | Know. index | Self-decl. energy know. | Trust | Economic hardship | Climate resp. | Covid resp. | Prec. | Adj. R2 |
| --- | --- | --- | --- | --- | --- | --- | --- | --- | --- | --- | --- | --- | --- |
| *Australia (n=1827)* | |  |  |  |  |  |  |  |  |  |  |  |  |
| Climate sceptics & antivaxxers | 2.96E-5 | | 1.40E-3 | -0.01* | -2.56E-3 | 2.58E-3 | -1.77E-5 | -3.10E-3** | 1.11E-3 | -6.87E-4 | -2.55E-4 | 2.42E-4 | 0.27 |
| Climate sceptics only | 4.48E-4** | | -0.01* | -0.06*** | -0.01T | -3.00E-3* | 4.24E-3* | -0.01** | -1.78E-3 | -3.65E-3** | -8.65E-3 | -1.66E-3* | 0.28 |
| Antivaxxers only | -3.70E-4T | | 2.90E-4 | -3.72E-3 | -0.01T | 5.70E-4 | -3.60E-3 | -0.01*** | 0.01* | 4.93E-3** | -8.72E-4 | 2.90E-3* | 0.11 |
| *Brazil (n=1825)* |  |  |  |  |  |  |  |  |  |  |  |  |  |
| Climate sceptics & antivaxxers | 2.14E-5 | | -3.61E-3T | X | -4.70E-3 | 1.33E-4 | 1.53E-3T | -1.70E-3* | -2.64E-4 | -2.37E-4 | -9.44E-4* | -3.34E-4 | 0.33 |
| Climate sceptics only | 3.96E-4T | | -0.01* | -0.03*** | -0.01 | -1.12E-3 | 3.17E-4 | -3.17E-3T | 9.01E-4 | 1.04E-4 | -1.21E-3 | -1.77E-3T | 0.08 |
| Antivaxxers only | 3.86E-4T | | -0.01 | -6.90E-4 | -0.01 | 9.82E-4 | 4.31E-3 | -0.01*** | 4.35E-3 | -1.65E-3 | 1.19E-3 | 1.24E-3 | 0.07 |
| *China (n=1543)* |  |  |  |  |  |  |  |  |  |  |  |  |  |
| Climate sceptics & antivaxxers | 7.95E-5 | | X | X | -1.23E-3 | -2.76E-4 | -5.16E-4 | -7.30E-5 | -1.36E-3 | 1.32W-4 | 1.04-e4 | 4.29E-4 | 0.20 |
| Climate sceptics only | 6.21E-4** | | 6.23E-4 | -0.01T | -4.12E-3 | -0.01** | -4.12E-3 | -3.01E-3 | -6.96E-5 | 0-2.80E-3 | -0.01** | -3.25E-4 | 0.19 |
| Antivaxxers only | 8.48E-5 | | -3.16E-3 | -3.55E-5 | 1.83E-3 | 1.12E-3T | 4.77E-4 | -2.68E-4 | 8.82E-4 | 2.45E-6 | 2.80E-4 | -6.701E-5 | 0.10 |
| *UK (n=1866)* |  |  |  |  |  |  |  |  |  |  |  |  |  |
| Climate sceptics & antivaxxers | -3.75E-5 | | 1.43E-3 | -1.18E-3 | -9.99E-4 | 3.50E-4 | 4.10E-4 | -9.89E-4T | 7.70E-4 | -1.27E-4 | -1.21E-4 | 7.42E-6 | 0.23 |
| Climate sceptics only | 7.57E-5 | | -0.01* | -0.02*** | -0.01 | -1.13E-3 | 1.98E-3 | -0.01*** | 1.57E-3 | -2.13E-3* | 1.76E-4 | -7.29E- | 0.23 |
| Antivaxxers only | -1.11E-4 | | 4.30E-3 | 1.52E-3 | -0.01 | -2.67E-3T | -7.09E-4 | -0.01** | 4.20E-3 | 2.96E-3T | -5.90E-4 | 1.17E-3 | 0.07 |
| *India (n=1880)* |  |  |  |  |  |  |  |  |  |  |  |  |  |
| Climate sceptics & antivaxxers | -2.68E-6 | | -1.19E-4 | 6.48E-4 | X | -5.17E-5 | -1.31E-4 | -6.71E-5 | -1.39E-5 | 3.84E-5 | 1.17E-5 | 1.23E-5 | 0.35 |
| Climate sceptics only | -4.48E-5 | | -0.01* | -0.01T | -0.02* | -1.00E-3 | -0.01T | -4.66E-3* | -7.43E-4 | -1.84E-3 | 9.00E-4 | 5.32E-4 | 0.08 |
| Antivaxxers only | 1.06E-4 | | -0.01 | -0.01 | -0.01 | 0.01* | -3.56E-3 | -4,00E-4 | 0.01*** | 4,21E-4 | 1.83E-3 | 2.33E-3* | 0.05 |
| *Japan (n=1665)* |  |  |  |  |  |  |  |  |  |  |  |  |  |
| Climate sceptics & antivaxxers | 1.82E-6 | | -2.14E-3 | -3.83E4 | -1.20E-4 | -3.56E-4 | 4.45E-5 | -1.39E-2* | -4.26E-4 | 2.53E-4 | 3.02E-4 | 2.87E-4 | 0.27 |
| Climate sceptics only | -4.20E-5 | | -0.03** | -0.03*** | -0.01 | -0.01** | 0.01** | -3.76E-3 | -4.91E-3 | 1.72E-3 | -1.45E-3 | -9.51E-4 | 0.08 |
| Antivaxxers only | -4.01E-4** | | 4.22E-3 | -0.01T | -1.54E-3 | -2.46E-3* | 2.52E-3 | -0.01*** | -2.35E-5 | 9.28E-4 | -4.41E-4 | 1.99E-3* | 0.12 |
| *South Africa (n=1899)* |  |  |  |  |  |  |  |  |  |  |  |  |  |
| Climate sceptics & antivaxxers | 3.10E-4** | | -0.01 | X | -0.01T | -8.80E-4 | 9.19E-4 | -4.82E-3*** | -1.38E-3 | -1.94E-4 | -8.34E-4 | 7.62E-4 | 0.16 |
| Climate sceptics only | 1.27E-4 | | -2.72E-3 | -0.01 | -0.01 | -2.80E-3 | -5.55E-4 | -3.93E-3* | -6.93E-4 | 3.97E-4 | 1.12E-3 | -1.10E-3 | 0.05 |
| Antivaxxers only | 4.93E-4 | | 0.04* | -0.01 | -3.01E-3 | -1.19E-4 | 3.12E-3 | -0.03*** | 0.04*** | 2.21E-3 | -6.70E-4 | 0.01* | 0.06 |
| *US (n=1823)* |  |  |  |  |  |  |  |  |  |  |  |  |  |
| Climate sceptics & antivaxxers | -3.54E-5 | | -5.67E-4 | X | -0.04*** | -1.63E-3 | 3.14E-3 | -0.01*** | -3.90E-4 | 1.17E-3 | -0.01*** | 1.67E-4 | 0.31 |
| Climate sceptics only | 7.74E-4*** | | -0.02** | -0.06*** | -0.02** | -0.01** | 3.81E-3 | -0.01*** | 1.82E-3 | -0.01** | 8.91E-4 | -1.61E-3 | 0.29 |
| Antivaxxers only | -8.76E-4*** | | 0.02** | -0.01 | -0.01 | -1.16E-3 | -1.82E-3 | -0.01*** | 0.01T | 3.32E-3T | -2.44E-3 | -2.00E-3 | 0.10 |

Table S18: Probability of being a double sceptic, climate sceptic or antivaxxer across countries.

Note: The dependent variable is binary, taking the value of 1 if an individual response is categorized as the defined sceptic attitude towards climate change and COVID-19 and 0 otherwise. For each country, Row 1 estimates the probability of an individual being both climate sceptic and antivaxxer, Row 2 climate sceptic but not antivaxxer and Row 3 antivaxxer but not climate sceptic. Country regressions include (unreported) education variables that denote the core distinctions between levels of educational attainment in each country as specified in Supplementary Tables 3-10. T denotes P<0.10, *P<0.05, **P<0.01 and ***P<0.001.

| Country | Age | Female | Prioritise env. | Prioritise health | Know index | Self-decl. energy know. | Trust | Perceived income sufficiency | Climate resp. | Covid resp. | Prec. | Adj. R2 |  |
| --- | --- | --- | --- | --- | --- | --- | --- | --- | --- | --- | --- | --- | --- |
| *Australia (n=1827)* | | | | | | | | | | | | | |
| Econ. Over climate & COVID-19 | -7.77E-5 | -1.45E-3 | -4.79E-3T | -0.01* | -8.11E-4 | 2.47E-3* | -1.67E-3* | -5.83E-4 | -1.05E-3* | -6.70E-4 | -6.67E-4 | 0.23 |  |
| Econ. over climate only | 8.46E-4*** | -0.01 | -0.05*** | -0.01 | -4.39E-3** | 0.01T | -0.01** | -1.30E-3 | -3.63E-3* | 3.16E-3* | 3.91E-5 | 0.18 |  |
| Econ. Over COVID-19 only | -1.10E-4 | -1.00E-3 | 1.89E-3 | -0.01T | -1,51E-3 | 4.16E-3** | -1.39E-3 | 1.33E-3 | -1.92E-3 | -1.13E-3T | 9.29E-4 | 0.14 |  |
| *Brazil (n=1825)* | | | | | | | | | | | | | |
| Econ. Over climate & COVID-19 | -2.95E-5 | -2.74E-3 | -0.03*** | -0.01T | -1.12E3 | 3.80E-3* | -3.36E-3** | 7.59E-4 | -2.18E-3* | -1.41E-3T | 2.70E-3** | 0.16 |  |
| Econ. over climate only | 2.07E-4 | -0.01 | -2.37E-3 | -7.11E-6 | -0.01** | 0.01 | -0.01* | 0.01 | -1.53E-3 | 2.26E-3 | 0.01*** | 0.07 |  |
| Econ. Over COVID-19 only | 2.13E-4 | 1.16E-3 | -0.01 | -1.62E-3 | -1.58E-3 | 3.76E-3 | -2.18E-3 | -2.00E-3 | -1.26E-3 | -0.01*** | -2.32E-4 | 0.11 |  |
| China (n=1571) | | | | | | | | | | | | | |
| Econ. Over climate & COVID-19 | 2.65E-6 | -3.22E-4 | -1.93E-4 | -1.67E-4 | -2.09E-4 | 1.32E-4 | 8.36E-5 | -1.11E-4 | -1.50E-4 | 1.31E-6 | 8.54E-5 | 0.45 |  |
| Econ. over climate only | -2.09E-5 | 3.27E-3 | -0.01* | -1.57E-3 | -1.07E-3 | 3.45E-3 | -4.44E-4 | 0.01* | 1.44E-3 | 1.02E-3 | 2.56E-3** | 0.09 |  |
| Econ. Over COVID-19 only | 1.11E-4T | -6.90E-4 | -1.00E-3 | -7.27E-5 | -7.40E-4 | 4.56E-4 | -8.72E-4 | 2.60E-3* | 2.79E-4 | -2.35E-5 | 1.57E-3** | 0.19 |  |
| *UK (n=1866)* | | | | | | | | | | | | | |
| Econ. Over climate & COVID-19 | -6.48E-5 | -3.78E-3 | -4.93E-3 | -3.37E-3 | -4.06E-4 | 2.01E-3T | -2.34E-3** | -4.54E-6 | -7.03E-4 | -1.46E-4 | -5.33E-6 | 0.21 |  |
| Econ. over climate only | 5.71E-4** | -4.93E-3 | -0.04*** | -0.01 | -3.10E-3* | -4.45E-3 | -2.68E-3T | 1.23E-4 | -1.24E-4 | -3.53E-4 | 2.90E-3** | 0.15 |  |
| Econ. Over COVID-19 only | -8.61E-5 | 1.07E-3 | 1.44E-3 | -0.01** | 2.69E-4 | 1.19E-5 | -1.45E-3* | 2.66E-4 | 3.78E-4 | -6.98E-4T | 1.73E-5 | 0.23 |  |
| *India (n=1880)* | | | | | | | | | | | | | |
| Econ. Over climate & COVID-19 | 1.91E-4 | 3.95E-3 | -0.01 | -0.01T | -3.70E-3* | 0.01*** | 1.11E-3 | 1.58E-3 | -2.61E-3** | -2.80E-3** | 0.01*** | 0.31 |  |
| Econ. over climate only | -1.46E-5 | 0.02T | -0.02T | 0.01 | -0.01** | -7.29E-6 | 0.01T | 0.01** | 1.22E-3 | -4.15E-4 | 0.01*** | 0.06 |  |
| Econ. Over COVID-19 only | -1.62E-4 | -0.01 | 0.01 | -0.01 | -1.57E-3 | 1.24E-3 | -1.11E-3 | 2.65E-4 | -1.79E-3 | -8.02E-4 | 4.40E-3*** | 0.05 |  |
| *Japan (n=1773)* | | | | | | | | | | | | | |
| Econ. Over climate & COVID-19 | -5.24E-5 | -1.90E-3 | 1.-3.90E-3 | 2.33E-3 | -1.08E-3* | 2.70E-3* | -1.98E-3* | -4.10E-5 | 1.10E-4 | 2.56E-4 | 5.24E-4 | 0.21 |  |
| Econ. over climate only | -2.97E-5 | -0.01** | -0.02* | -0.01 | -1.92E-3T | 4.22E-3* | 6.79E-4 | 1.75E-4 | 1.97E-3T | 1.50E-3T | 2.22E-3** | 0.15 |  |
| Econ. Over COVID-19 only | -1.12E-4T | 1.34E-3 | -1.00E-3 | 2.90E-3 | -1.77E-4 | -9.13E-6 | -9.01E-4 | 9.19E-4T | -6.03E-4 | -2.35E-4 | 7.86E-4T | 0.17 |  |
| *South Africa (n=1871)* | | | | | | | | | | | | | |
| Econ. Over climate & COVID-19 | 4.01E-4T | 3.37E-3 | -0.01 | -4.60E-3 | -1.48E-3 | -1.48E-3 | -0.01*** | -1.72E-3 | 5.52E-3 | -3.04E-3** | 0.01*** | 0.10 |  |
| Econ. over climate only | -4.56E-4 | 0.01 | -3.20E-3 | -0.01 | -0.02*** | 0.01** | 4.72E-4 | 0.02** | 0.01*** | -2.40E-3 | 4.88E-3** | 0.07 |  |
| Econ. Over COVID-19 only | 6.61E-4* | 0.01 | -0.01 | -0.03** | 0.01** | 2.04E-3 | -0.01* | 0.01* | -1.40E-3 | -3.80E-3* | 2.03E-3 | 0.05 |  |
| *US (n=1862)* | | | | | | | | | | | | | |
| Econ. Over climate & COVID-19 | -1.66E-5 | -0.01 | X | -0.02** | -5.91E-4 | 4.88E-3T | -0.01*** | -2.04E-3 | 2.689E-5 | -0.01*** | 2.07E-3* | 0.38 |  |
| Econ. over climate only | 7.78E-4*** | -4.01E-5 | -0.05*** | -0.02** | -0.01** | 0.02*** | -3.91E-3T | 2.62E-3 | -0.01*** | 3.10E-3* | -7.99E-4 | 0.15 |  |
| Econ. Over COVID-19 only | -3.26E-4* | -2.15E-3 | -3.48E-3 | -0.01* | -0.02*** | 5.69E-4 | 3.43E-3 | -1.89E-3 | 8.30E-4 | 1.46E-3 | -2.22E-3* | 0.13 |  |

Table S19: Probability of giving complete priority to the economy over climate protection or protecting the public against COVID-19 or both.

Note: The dependent variable is binary, taking the value of 1 if an individual response is categorized as the defined sceptic attitude towards climate change and COVID-19 and 0 otherwise. For each country, Row 1 shows the probability of an individual giving complete priority to the economy over combatting climate change and the pandemic, Row 2 the probability of giving complete priority to the economy over climate protection but not combatting COVID-19 and Row 3 the probability of giving complete priority to the economy over combatting COVID-19 but not climate protection. Country regressions include (unreported) education variables that denote the core distinctions between levels of educational attainment in each country as specified in Supplementary Tables 3-10. T denotes P<0.10, *P<0.05, **P<0.01 and ***P<0.001.

**Operationalisation and Coding**

**Antivaxxers and climate sceptics**

Scepticism towards COVID-19 vaccination and climate change were inferred from respondent ratings of their willingness to take a COVID-19 vaccination if offered one and the level of threat that climate change poses to their country, respectively. As specified in Table S2, our primary focus is the subset of national publics who hold the most extreme sceptical perspective towards COVID vaccination and climate change. Since the degree of scepticism that individuals can feel towards a given issue can span a spectrum, Likert scale responses were used to allow respondents to rate their level of level scepticism towards each issue separately. These responses were subsequently dichotomized to indicate whether respondents exhibited the most sceptical stance towards each domain. Moreover, since substantial shares of scepticism ratings were in the middle, rather than the extremes of the Likert scales, using binary response options (e.g. climate change is/ is not a threat to my country or I would / would not be willing to take the vaccine) instead of more graduated Likert scales would likely have distorted data collection by forcing participants to choose from two extreme scepticism ratings.

**Prioritisation of the environment and health**

Past research suggests that climate concern is inversely associated with climate scepticism.^[[1]](#endnote-2)^ Although antivaxxism has been linked to scepticism over the benefits and adverse consequences of vaccination rather than a lack of concern over public health^[[2]](#endnote-3)^, scepticism towards COVID-19 vaccination could be influenced by assessment of the priority of COVID-19 mitigation relative to other priorities such as the economy. Therefore, we included binary variables to account for whether people rated the environment/ public health as a top-three national priority (1) or otherwise (0) out of the following issues: health, immigration, crime, the economy, tax, pensions, education, family life & childcare, international relations, the environment and transport.

**Knowledge**

It is well established that science knowledge is inversely associated with climate scepticism^[[3]](#endnote-4),^^[[4]](#endnote-5)^ and controversial science topics more generally.^[[5]](#endnote-6)^ Recent studies suggest that scientific understanding is also negatively associated with hesitancy towards COVID-19 vaccination.^[[6]](#endnote-7)^ Our survey data provide a range of different options for measuring science knowledge such as self-declared knowledge about different energy technologies and factual questions regarding a range of energy, environmental and health science knowledge questions. Preliminary analyses (not reported) revealed that two measures in particular were most significantly associated climate scepticism and antivaxxism, prompting us to include two variables to account for energy knowledge in our models. First, we asked respondents to rate their knowledge about how energy is produced, delivered and used on a five-point scale from 1 (not at all knowledgeable) to 5 (very knowledgeable). Second, we asked respondents to describe their views towards a battery of eight energy and environment statements used to evaluate objective knowledge about energy and environmental science: (i) ‘we are currently in a warm period between ice ages’, (ii) ‘roughly two thirds of the energy used to produce electricity from fossil fuels is lost’, (iii) ‘climate change is caused by a hole in the earth’s atmosphere’, (iv) ‘every time we use coal or oil or gas, we contribute to climate change’, (v) ‘oil and gas reservoirs are typically found 100 meters below the surface’, (vi) ‘oxygen is the main component of the smoke emitted from a chimney or exhaust pipe’, (vii) ‘dinosaurs were alive 1 million years ago’, (viii) ‘coal is produced from dead plants’ and ‘viruses can be treated with antibiotics’. Answer options ranged from 1 (confident this is true) to 4 (confident that this is false) and included an additional ‘don’t know’ option. Confident correct responses were coded as correct responses (1) and all other responses were coded as incorrect (0), and merged in an aggregate knowledge index ranging from 0 to 9 correct scores. As an alternative approach, we accounted for confidence in responses to energy knowledge questions by coding confident correct responses as 1, unconfident correct responses as 0.5, unconfident incorrect responses as -0.5, confident incorrect responses as -1 and ‘don’t know’ responses as 0, which were then merged in an aggregate confident knowledge index ranging from -8 to 8 and found that the estimated associations between knowledge, trust in scientists and the probability of being a ‘double’ or single sceptic were largely the same whether we used the pure knowledge or confidence-sensitive knowledge index (see Supplementary Tables 18-19).

**Covid/ climate responsibility**

Attitudes towards major societal problems such as climate change have been associated with the relative level of responsibility that one attributes to individuals versus governments for resolving such problems.^[[7]](#endnote-8)^ Therefore, we accounted for potential confounding by asking respondents how much responsibility, if any, individuals had in (separately) solving climate change and COVID-19 compared to other institutions (e.g. governments, business and charities). Responses were on an eleven-point scale from 0 (completely the responsibility of other institutions) to 10 (completely the responsibility of individuals).

**Precautionism**

Prior research suggests that individuals who are generally supportive of precautionary measure for pre-empting potentially serious societal problems tend to be more supportive of climate mitigation and, potentially, precautionary medical practices such as vaccination.^[[8]](#endnote-9),^^[[9]](#endnote-10)^ We therefore accounted for precautionary preferences by asking respondents whether they felt, in general, that is it better to take action in anticipation of what may become a serios problem based on uncertain information or wait to see if the problem develops into a serious problem and take action then ? Responses were on an eleven-point Likert scale from 0 (definitely take action immediately) to 10 (definitely wait and see).

**Demographics**

Age was recorded in years using an open-ended question. Gender was recorded in binary format in Brazil, China, India, Japan and South Africa and a non-binary option was included in Australia, the UK and US. Given the usually high non-response rate to direct income questions and the difficulty of comparing incomes across countries, respondents were asked to describe their current household financial situation by selecting one of the following options: living comfortably on present income, coping on present income, finding it difficult on present income, finding it very difficult on present income, prefer not to say and don’t know. We also asked respondents to indicate the highest level of education they had completed from country-specific educational categories (see Supplementary Tables 3-10). There are many country-specific differences in education systems, so responses were dichotomised as undergraduate degree (1) and lower level of education (0) to facilitate comparison in the pooled models.

**Table S20 Hierarchical model showing relative log odds associated with different sceptic profiles**

| **Parameter** | **Climate sceptic** | **Antivaxxer** | **Double sceptic** |
| --- | --- | --- | --- |
| *Fixed effects* | | | |
| Age | 0.02*** | -0.01** | 2.22E-4 |
| Female | -0.77*** | 0.17T | -0.17 |
| Degree | -0.01 | -0.27** | -0.36T |
| Prioritise environment | -1.90*** | -0.15 | -1.66** |
| Prioritise health | -0.40*** | -0.20* | -0.85*** |
| Objective knowledge | -0.15*** | 3.60E-3 | -0.03 |
| Self-declared energy knowledge | 0.04 | 3.70E-3 | 0.16T |
| Perceived income insufficiency | -0.04 | 0.24*** | 0.21* |
| Climate responsibility | -0.11*** | 0.06** | 0.01 |
| COVID-19 responsibility | 0.04T | -0.01 | -0.17*** |
| Precautionism | -0.05** | 0.04* | 0.01 |
| Trust government | -0.03 | -0.14*** | -0.10*** |
| Trust oil and gas comp. | 0.24** | 0.01 | 0.05 |
| Trust television | -0.20*** | -0.21*** | -0.62*** |
| Trust scientists | -0.27*** | -0.18*** | -0.34*** |
| Left-right orientation | 0.08*** | 0.05** | 0.11** |
| *Random effects* | | | |
| Country variance | 0.22*** | 0.39*** | 0.34*** |
| R2 equivalent | 0.01 | 0.56 | 0.70 |
| LR test | 54.91*** | 188.89*** | 11.54*** |
| N | 9992 | 9992 | 9992 |

Table S20: Effects on the relative log odds of being a climate sceptic, antivaxxer or both in hierarchical configurations.

Note: The dependent variable is binary, taking the value of 1 if an individual response is categorized as fitting the specified sceptic profile and 0 otherwise. Models are two-level hierarchical logistic regressions (individuals nested in countries) with random intercepts fitted using Stata’s xtmelogit command. In accordance with Snijders and Bosker (1994),^[[10]](#endnote-11)^ (country level) null variance components were used to calculate the percentage of explained variance at the country level and reported as the R2 equivalent. T denotes P<0.10, *P<0.05, **P<0.01 and ***P<0.001.

**Supplementary Tables S21-20: Post hoc power evaluations**

| Power | Double sceptics | Climate sceptics | Antivaxxers |
| --- | --- | --- | --- |
| 0.60 | 544 | 1409 | 1189 |
| 0.70 | 645 | 1716 | 1446 |
| 0.80 | 687 | 2112 | 1779 |
| 0.90 | 804 | 2730 | 2298 |
| R2 | 0.27 | 0.16 | 0.14 |
| Actual N (pooled = 14956, country samples = 1543 to 1899) | | | |

Table S21: Sample sizes required for core specifications (summarised in Table 3) to obtain selected power levels (0.6 to 0.9).

Note: Alpha = 0.01, R2 is set to the estimated R2 values from the main specifications (Table 3) using probabilities computed at trust in scientists = 1 and 2.

**Power sensitivity analysis**

| **Parameter** | **Double sceptic** | **Climate sceptic** | **Antivaxxer** |
| --- | --- | --- | --- |
| Age | -2.01E-5 | 8.80E-4*** | -3.00E-4* |
| Female | -1.90E-3 | -0.05*** | 0.01* |
| Degree | -0.01** | -0.01 | -0.02*** |
| Prioritise environment | -0.03*** | -0.20*** | -0.02*** |
| Prioritise health | -0.02*** | -0.03** | -0.03*** |
| Objective knowledge | -7.89E-4 | -0.01*** | -1.04E-3 |
| Self-declared energy knowledge | 3.07E-4 | -0.01T | 7.89E-5 |
| Perceived income insufficiency | 3.20E-3** | -0.03*** | 0.02*** |
| Climate responsibility | 6.70E-4 | -0.01*** | 2.14E-3* |
| COVID-19 responsibility | -2.40E-3*** | -1.90E-3 | -2.87E-3** |
| Precautionism | -6.45E-4T | -0.01*** | 8.74E-4 |
| Trust scientists | -0.01*** | -0.03*** | -0.02*** |
| R2 equivalent | 0.17 | 0.09 | 0.15 |
| No. positive outcomes | 866 | 4751 | 1901 |
| N | 14956 | 14956 | 14956 |

Table S22: Effect sizes of key variables on the probability that an average individual is a: (A) climate sceptic, antivaxxer or both (N=14956) using broader sceptic profiles.

Note: The dependent variable is binary, taking the value of 1 if an individual response is categorised as the defined sceptic attitude towards climate change and COVID-19 and 0 otherwise. Respondents who answered that climate change is ‘not a threat’ or ‘a minor threat to their country’ are coded as climate sceptics, respondents who were ‘very unlikely’ or ‘would definitely not’ take a COVID-19 vaccine were coded as antivaxxers. Respondents who fit both antivaxxer and climate sceptic profiles were coded as double sceptics. Model 1A estimates the probability of an individual being both climate sceptic and antivaxxer, model 2A climate sceptic but not antivaxxer and model 3A antivaxxer but not climate sceptic. . Coefficients are marginal effect sizes that describe the probability that an average individual holds a specified sceptic (or associated economy-prioritizing) profile as a result of a change in the independent variable. Marginal effect sizes are based on conversations of the effects of the independent variables on the relative log odds of the sceptic profiles (log odds results are available on request). Country controls are included but not reported. ‘No. of positive outcomes’ denotes number of respondents who possess the sceptic attitude (or corresponding prioritisation of the economy) captured in the dependent variable. *P<0.05, **P<0.01 and ***P<0.001. In this study, all reported R2 values are McFadden Pseudo R2s unless stated otherwise.

**Supplementary Table S23:Summary sceptic samples across separate country samples and pooled sample**

|  | **Australia** | **Brazil** | **China** | **UK** | **India** | **Japan** | **S Africa** | **US** | **Pooled** |
| --- | --- | --- | --- | --- | --- | --- | --- | --- | --- |
| **Attitudes towards climate change** | | | | | | | | |  |
| A major threat | 1118 | 1440 | 880 | 1240 | 1440 | 1262 | 1340 | 1040 | 9760 |
| A minor threat | 585 | 400 | 1000 | 600 | 420 | 509 | 520 | 600 | 6634 |
| Not a threat | 189 | 100 | 80 | 80 | 80 | 101 | 80 | 280 | 990 |
| Don’t know | 107 | 60 | 40 | 80 | 60 | 163 | 60 | 80 | 650 |
| **Attitudes towards COVID vaccine** | | | | | | | | |  |
| Already taken it/ would definitely take it | 690 | 1240 | 800 | 1420 | 840 | 265 | 440 | 920 | 6615 |
| Very likely to take it | 378 | 220 | 580 | 220 | 460 | 488.4 | 238 | 260 | 2844 |
| Somewhat likely to take it | 276 | 160 | 300 | 120 | 240 | 531 | 238 | 190 | 2055 |
| Neither likely nor unlikely to take it | 216 | 80 | 200 | 60 | 144 | 330 | 238 | 114 | 1382 |
| Somewhat unlikely to take it | 120 | 80 | 60 | 40 | 84 | 167 | 166 | 114 | 831 |
| Very unlikely to take it | 120 | 80 | 40 | 40 | 104 | 138 | 240 | 154 | 916 |
| Would definitely not take it | 120 | 100 | 20 | 60 | 84 | 63 | 400 | 200 | 1047 |
| Don't know | 80 | 40 | 0 | 40 | 42 | 53 | 40 | 48 | 343 |
| Total no. respondents in sample | 2000 | 2000 | 2000 | 2000 | 2035 | 2000 | 2000 | 2000 | 16035 |

Supplementary Table S23: Summary climate sceptic and antivaxxer statistics across country and pooled samples.

**Methods References**

1. Tranter, B. & Booth, K. Scepticism in a changing climate: A cross-national study. *Global Environmental Change* **33**, 154–164. [↑](#endnote-ref-2)
2. Hornsey, M. J., Edwards, M., Lobera, J., Diaz-Catalan, C. & Barlow, F. K. Resolving the small-pockets problem helps clarify the role of education and political ideology in shaping vaccine scepticism. *Br J Psychol* (2021) doi:10.1111/bjop.12500. [↑](#endnote-ref-3)
3. Hobman, E. V & Ashworth, P. Public support for energy sources and related technologies: The impact of simple information provision. *Energy Policy* **63**, 862–869 (2013). [↑](#endnote-ref-4)
4. Turaga, R. M. R., Howarth, R. B. & Borsuk, M. E. Pro-environmental behavior. *Ann N Y Acad Sci* **1185**, 211–224 (2010). [↑](#endnote-ref-5)
5. Drummond, C. and Fischhoff, B. Individuals with greater science literacy and education have more polarized beliefs on controversial science topics. PNAS 114, 36 (2017). [↑](#endnote-ref-6)
6. Brzezinski, A., Kecht, V., Van Dijcke, D. & Wright, A. *Belief in Science Influences Physical Distancing in Response to COVID-19 Lockdown Policies* (2020). [↑](#endnote-ref-7)
7. Rickard, L. N., Yang, Z. J., Seo, M. & Harrison, T. M. The “I” in climate: The role of individual responsibility in systematic processing of climate change information. *Global Environmental Change* **26**, 39–52 (2014). [↑](#endnote-ref-8)
8. Sunstein, C. R. Beyond the Precautionary Principle. *University of Pennsylvania Law Review* **151**, 1003 (2003). [↑](#endnote-ref-9)
9. Sadeleer, N. De. The Precautionary Principle in EC Health and Environmental Law. *European Law Journal* **12**, 139–172 (2006). [↑](#endnote-ref-10)
10. Snijders, T., & Bosker, R. (1994). Modelled variance in two-level models. Sociological Methods and Research, 22(3), 342–363. [↑](#endnote-ref-11)
